# Plasmid Replicons from *Pseudomonas* Are Natural Chimeras of Functional, Exchangeable Modules

**DOI:** 10.3389/fmicb.2017.00190

**Published:** 2017-02-13

**Authors:** Leire Bardaji, Maite Añorga, José A. Ruiz-Masó, Gloria del Solar, Jesús Murillo

**Affiliations:** ^1^Departamento de Producción Agraria, Escuela Técnica Superior de Ingenieros Agrónomos, Universidad Pública de NavarraPamplona, Spain; ^2^Molecular Biology of Gram-Positive Bacteria, Molecular Microbiology and Infection Biology, Centro de Investigaciones Biológicas (Consejo Superior de Investigaciones Científicas)Madrid, Spain

**Keywords:** control and replication modules, chimeric replicons, gene co-option, Rep proteins, origin of replication, plasmid incompatibility, swapping of functional modules, virulence plasmids

## Abstract

Plasmids are a main factor for the evolution of bacteria through horizontal gene exchange, including the dissemination of pathogenicity genes, resistance to antibiotics and degradation of pollutants. Their capacity to duplicate is dependent on their replication determinants (replicon), which also define their bacterial host range and the inability to coexist with related replicons. We characterize a second replicon from the virulence plasmid pPsv48C, from *Pseudomonas syringae* pv. savastanoi, which appears to be a natural chimera between the gene encoding a newly described replication protein and a putative replication control region present in the widespread family of PFP virulence plasmids. We present extensive evidence of this type of chimerism in structurally similar replicons from species of *Pseudomonas*, including environmental bacteria as well as plant, animal and human pathogens. We establish that these replicons consist of two functional modules corresponding to putative control (REx-C module) and replication (REx-R module) regions. These modules are functionally separable, do not show specificity for each other, and are dynamically exchanged among replicons of four distinct plasmid families. Only the REx-C module displays strong incompatibility, which is overcome by a few nucleotide changes clustered in a stem-and-loop structure of a putative antisense RNA. Additionally, a REx-C module from pPsv48C conferred replication ability to a non-replicative chromosomal DNA region containing features associated to replicons. Thus, the organization of plasmid replicons as independent and exchangeable functional modules is likely facilitating rapid replicon evolution, fostering their diversification and survival, besides allowing the potential co-option of appropriate genes into novel replicons and the artificial construction of new replicon specificities.

## Introduction

Plasmids are extrachromosomal elements that colonize a vast majority of bacteria and other organisms, often carrying genes that confer an adaptive advantage to the host (del Solar et al., 1998; Jackson et al., 2011; Ruiz-Masó et al., 2015). Each cell can have from none to several plasmids of diverse sizes and copy numbers. Plasmids can readily acquire large amounts of foreign DNA from different sources and are transferred between distantly related organisms, including prokaryotes and eukaryotes, which makes them a major contributor to the accessory gene pool and the most important agents in horizontal gene transfer (Halary et al., 2010; Jackson et al., 2011). Indeed, plasmids are responsible for the worldwide distribution of genes for resistance to antibiotics and other antimicrobials, rendering current strategies ineffective for the control of human, animal and plant diseases (Sundin, 2007; Jackson et al., 2011; Aviv et al., 2016; Johnson et al., 2016).

The basic replicon is the fundamental element for plasmid survival, ensuring timely duplication in coordination with cell division (Nordström, 1993; Summers, 1996; del Solar et al., 1998). Broadly, basic replicons consist of (i) a short *cis*-acting DNA sequence, the origin of replication, (ii) genes and structures involved in the control of replication and, for most plasmids, (iii) a gene coding a replication initiator (Rep) protein that recognizes the origin and promotes initiation of DNA replication. Plasmid replication is controlled by either directly repeated sequences (iterons) or by antisense RNAs, which can act alone or in coordination with a protein repressing transcription of the *rep* gene, and is tightly regulated so as to maintain the number of plasmid molecules in the cell within acceptable limits (Summers, 1996; del Solar and Espinosa, 2000). An immediate consequence of this is that plasmids sharing elements for replication or replication control cannot coexist in the same cell and are hence incompatible (Novick, 1987).

Replicons are highly diverse and can be grouped based on their general mechanism of replication, the function of their Rep proteins, their structure and genetic organization or their homology (del Solar et al., 1998; Lilly and Camps, 2015). Circular plasmids replicate by one of three general modes: rolling-circle, strand-displacement and theta-type mechanisms. According to their mode of replication initiation, the theta-type replicons have been grouped into four classes (A, B, C, and D) (Bruand et al., 1993). Class A theta replicons (e.g., R1, RK2, R6K, pSC101, pPS10, F and P) encode a Rep protein that binds to the origin and mediates melting of the duplex DNA. Class B (ColE1-like) replicons lack a *rep* gene, and melting of duplex DNA as well as synthesis of a pre-primer RNA for replication are achieved by bacterial RNA polymerase-mediated transcription. Class C (ColE2- and ColE3-like) replicons contain the smallest origins reported so far and encode a Rep primase protein that also mediates unwinding of the DNA (Itou et al., 2015). Finally, functioning of class D replicons (plasmids pAMβ1, pIP501, and pSM19035 from Gram-positive bacteria) requires transcription across the origin and participation of a Rep protein in melting of the DNA and primer processing.

The gamma proteobacterial genus *Pseudomonas* comprises very diverse species, present in all kinds of environments, including significant human, animal and plant pathogens as well as species of outstanding biotechnological interest (Ramos, 2004). *Pseudomonas syringae* is one of the most relevant plant pathogenic bacteria in the world (Mansfield et al., 2012), and many strains carry one or more highly stable plasmids, ranging from a couple of kilobases to close to 1 Mb (Murillo and Keen, 1994; Sundin, 2007; Romanchuk et al., 2014). Most plasmids from *P. syringae*, and also various from many other *Pseudomonas* species, belong to the PFP (*p*PT23A-*f* amily *p*lasmid) group (Murillo and Keen, 1994; Sesma et al., 1998, 2000; Gibbon et al., 1999; Sundin, 2007). PFPs appear to originate from a common ancestor because they share homologous RepA-PFP replicons, which are related to the ColE2 class C theta replicons (Murillo and Keen, 1994; Sesma et al., 1998; Gibbon et al., 1999; Sesma et al., 2000; Sundin, 2007). ColE2 replicons contain a *rep* gene and an upstream region coding for a small antisense RNA, which is complementary to the 5′-nontranslated region of the *rep* mRNA and negatively controls its expression posttranscriptionally (Yasueda et al., 1994). Similarly, the RepA-PFP replicons consist of the *repA* replication initiator gene, which includes the putative vegetative origin of replication (Yagura et al., 2006), preceded by a short 5′ sequence, containing diverse stem-and-loop (SaL) structures, which is probably involved in control of replication (Murillo and Keen, 1994; del Solar et al., 1998; Gibbon et al., 1999; Brantl, 2014). PFP plasmids have had tremendous evolutionary success, not only for their ubiquity across pseudomonads, but also because most *P. syringae* strains contain two to six coexisting PFP plasmids (Murillo and Keen, 1994; Sesma et al., 1998). This could be explained in part because they generally carry genes essential for the interaction with the plant host or for survival, fostering their frequent exchange among the bacterial population (Sesma et al., 2000; Vivian et al., 2001; Ma et al., 2007; Sundin, 2007; Sundin and Murillo, 2009). Additionally, their competitiveness among the bacterial plasmid pool might be enhanced by a replication machinery particularly adapted to their bacterial host. Notwithstanding, the coexistence of PFP plasmids in the same cell is difficult to explain because of their potential incompatibility (Novick, 1987; Sesma et al., 1998). In fact, PFP plasmids are generally incompatible with their cloned replicons (Murillo and Keen, 1994; Murillo et al., 1994; Sesma et al., 1998) although subcloning did not allow for the identification of the sequences responsible for this incompatibility within the replicon (Gibbon et al., 1999).

*Pseudomonas syringae* pv. savastanoi NCPPB 3335 contains three virulence PFP plasmids, pPsv48A (78 kb), pPsv48B (45 kb), and pPsv48C (42 kb), of which the smallest two appear to have originated by plasmid duplication and reorganization (Bardaji et al., 2011). Plasmid pPsv48C is essential for elicitation of disease symptoms in the plant host olive (M. Añorga, unpublished results), and is extremely stable (Bardaji et al., 2011). In this work, we identified a second replicon on pPsv48C, designated here as RepJ replicon, containing a putative replication control region homologous to that of the pPsv48C RepA-PFP replicon (Bardaji et al., 2011). We also show that these, and structurally similar replicons, consist of two functional modules corresponding to the putative control region (REx-C module) and the replication region (REx-R module). These modules are functionally separable, do not show specificity for each other, and are dynamically exchanged among replicons of four distinct families. Additionally, a REx-C module from pPsv48C conferred replication ability to a non-replicative *repJ* chromosomal homolog. Thus, the organization of plasmid replicons as independent and exchangeable functional modules is likely fostering their diversification and survival, besides allowing the potential co-option of appropriate genes into novel replicons and the artificial construction of new replicon specificities.

## Results

### Definition of a second replicon in plasmid pPsv48C

**Figure 1:**
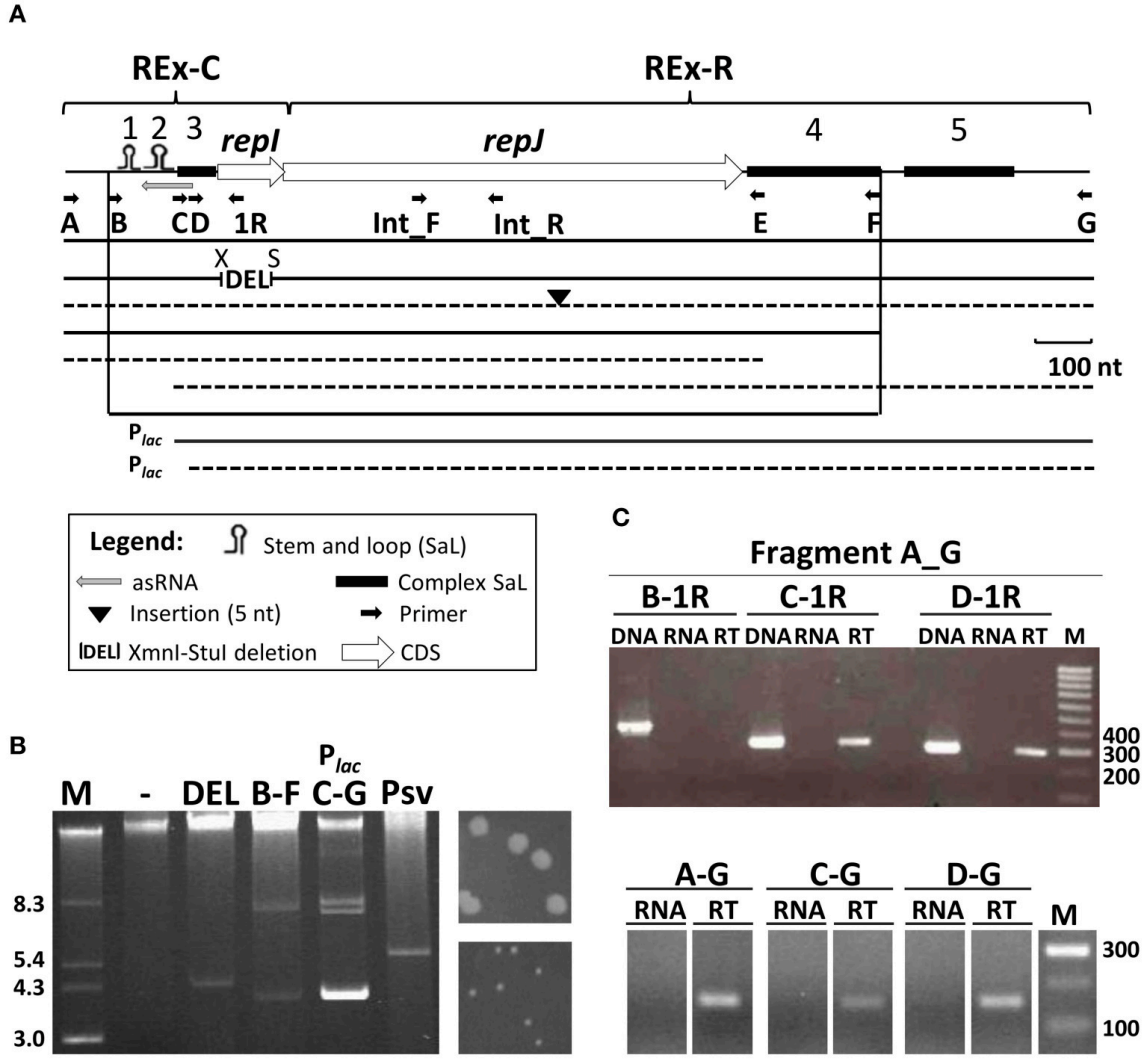
**Functional characterization of the RepJ replicon. (A)** The minimal replicon consists of two modules, REx-C and REx-R, whose extent is loosely defined here; stem-and-loop structures 1 to 5 are indicated by symbols (see legend) topped with the structure number; letters under small arrows indicate the primers used for amplification of fragments and for RT-PCR, and a gray arrow, the putative antisense RNA. Lines under the map indicate the relative size of fragments cloned after a transcriptional terminator in pKMAG, or downstream of the P_*lac*_ promoter in pK184. Continuous lines indicate constructs able to replicate autonomously in *P. syringae* pv. syringae B728a, and dashed lines indicate non-replicative fragments. X, XmnI; S, StuI. **(B)** Plasmid profiles of B728a transformants harboring constructs from panel A (indicated by letters) able to replicate and that define the minimal replicon; multiple bands correspond to different topological forms and/or multimers with the lowest bands corresponding to monomers and the top intense band to chromosomal DNA and high-molecular-weight plasmid multimers. Lane (-) is the wild type strain B728a and Psv is a derivative of NCPPB 3335 containing a 5.6 kb spontaneous deletion derivative of pPsv48C spanning the RepJ replicon. To the right, 48 h colonies of transformants containing constructs A–G (top) or C–G (bottom). **(C)** *repJ* is transcribed from at least two promoters. Upper gel: for RT-PCR, cDNA was synthesized from UPN912 containing construct A–G (in pKMAG) using the strand-specific primer Int_R, internal to *repJ*. Subsequent PCRs were done with primer pairs indicated above the gel and shown on **(A)**. Lower gel: for RT-PCR, cDNA molecules were synthesized from UPN912 containing constructs A–G, C–G, and D–G (the last two cloned in pKMAG-C) using the strand-specific primer Int_R. Further PCR amplification of cDNA was done using primer pair Int_F-Int_R, internal to *repJ* (see panel **A**). Amplifications were done with only DNA (DNA), only RNA (RNA) or cDNA (RT); M, molecular weight marker in kb (panel **B**) or nt (panel **C**).

In an independent study (M. Añorga, unpublished data), we observed the spontaneous generation of autonomously replicating deletion derivatives of pPsv48C lacking the RepA-PFP replicon (Figure 1B). The smallest derivative contains five putative coding sequences (CDSs), whose annotation is not related to plasmid replication, and a 661 nt fragment that appears in two nearly identical copies in pPsv48C (Figure S1). The second copy of this fragment precedes gene *repA*, matching the typical organization of RepA-PFP replicons, and was previously shown to be essential for replication of PFP plasmids (Murillo and Keen, 1994; Sesma et al., 1998; Gibbon et al., 1999; Sundin et al., 2004).

By cloning diverse PCR fragments into the *E. coli* vectors pK184 or pKMAG (Figure S2), which do not replicate in *Pseudomonas*, we determined that a 1,375 nt fragment (coordinates 29,386–30,760 in the pPsv48C sequence, accession no. FR820587; fragment B-F, Figure 1A and Figure S1), comprising around half of the 661 nt repeated fragment, contained all the essential elements for autonomous replication in the plasmidless strains *P. syringae* pv. syringae B728a (Figure 1B) and *P. syringae* pv. savastanoi UPN912. This fragment did not contain any obvious direct repetitions reminiscent of iterons, but was rich in palindromic structures and could adopt a complex folding structure. We could distinguish two well-defined structural regions in this minimal replicating fragment; based on their conservation and functionality (see below), we have defined these regions as plasmid *r*eplicon *ex*changeable (REx) modules: the Rex-C module contains the putative replication control system, whereas the Rex-R module comprises the replication system.

#### REx-C module

This is a 318 nt fragment (coordinates 29,386–29,703 in FR820587) that shows high identity to a fragment (coordinates 41,791-5) preceding the *repA* gene from pPsv48C and including its first two codons (Figure 1 and Figure S1). The fragment contributes the putative start codon, the promoter(s) and the RBS for the expression of the replication initiator gene *repJ* (see below). It also contains three SaL structures, the third of which is complex, potentially folding in different ways, and gene *repI*. By analogy with replicons lacking iterons (del Solar and Espinosa, 2000; Brantl, 2014), such as ColE2, this fragment also probably codes for a small antisense RNA, with a putative promoter within SaL 3 and for which SaL 1 could function as a transcription terminator (Figure S1). Replication assays with clones spanning partial fragments of the minimal replicon showed that deletion of SaL 1 and 2 abolished autonomous replication, although they were dispensable in clones maintaining the complex SaL 3 and the strong P*lac* promoter of the vector in the same transcriptional direction as *repJ* (fragment CG in Figure 1A). Nevertheless, bacteria transformed with construct CG required double the time than other replicative fragments to produce visible colonies (Figure 1B). Additionally, clones lacking the 5′ stem of SaL 3 did not sustain autonomous replication (fragment DG, Figure 1A and Figure S1), even when *repJ* was cloned in the transcriptional direction of the P*lac* promoter. These results likely suggest that expression of *repJ* from the P*lac* in this clone causes a lethal runaway replication phenotype (Nordström and Wagner, 1994) or that SaL 3 is also essential for replication.

Gene *repI* (PSPSV_C0037) is short (123 nt) and appears to be translationally coupled to the replication initiator gene *repJ*, which are characteristics of leader peptide genes needed for the control of replication of certain replicons (del Solar et al., 1998; Brantl, 2014). An XmnI-StuI in-frame deletion of 87 nt, spanning most of *repI* (Figure 1A and Figure S1) did not have any apparent effect in the replication ability of the RepJ replicon, indicating that the product of *repI* is not essential for replication and that spacing between the SaL structures and the start of gene *repJ* is flexible.

#### REx-R module

This module contains the replication initiator gene, *repJ* (PSPSV_C0038, 819 nt) and essential downstream sequences. The long, near-perfect ribosome binding site (5′-AAGGcGGTGA-3′) of *repJ* and its two first codons probably belong to the REx-C module, because they are part of a sequence highly conserved in the pPsv48C RepA-PFP replicon (Figure S1). Gene *repJ* is annotated as a putative transcriptional regulator and did not show significant homology to any domain in an InterPro search. However, the structure of 55 residues (residues 90–145) from RepJ could be modeled by Phyre2 with 81.6 % confidence, being similar to the N-terminal domain of a conserved replication initiator protein (Schumacher et al., 2014). Additionally, a construct containing a mutation causing a premature stop in *repJ* did not replicate in the plasmidless strains B728a and UPN912 (Figure 1). These results suggest that *repJ* codes for a replication initiator protein essential for autonomous replication.

After the *repJ* stop codon there is a *ca*. 0.5 kb fragment containing two blocks of repeated sequences that can form complex SaL structures, designated SaL 4 and 5, although only SaL 4 appears to be essential for autonomous replication (Figure 1A). Nevertheless, a blastn search with this fragment identifies sequences similar to SaL 5 situated 3′ of, among others, *rep* genes that are not homologous to *repJ*, such as those from plasmids pRA2 (from *P. alcaligenes* RA2), pP27494_2 (from *P. antarctica* PAMC 27494), pMBUI6 (from an uncultured bacterium), and pAOVO01 (from *Acidovorax* sp. JS42), and gene *krfA* from plasmid pTer331 (from *Colimonas fungivorans* Ter331).

### The REx-C module contains at least two active promoters

The absolute requirement of SaL 1 and 2 for replication can be overcome when *repJ* is transcribed from the strong P*lac* promoter, suggesting a plausible role of these structures in directing *repJ* transcription. We thus examined transcription of this gene by RT-PCR in clones lacking SaL 1, 2 and 3. Using clone AG in pKMAG (Figure 1C, upper gel), we observed a long *repJ* transcript that extended at least to the annealing site for primer C, but not to that for primer B. This indicates transcription from a promoter situated between the annealing sites for primers A and C (Figure S1), overlapping the putative antisense RNA, and possibly involved in transcription of *repI* and *repJ*. Amplification from smaller clones showed that *repJ* was transcribed even in the absence of SaL 1, 2 and 3 (fragments CG and DG, Figure 1C lower gel). Since the vectors used contain a T4 transcriptional terminator upstream of the cloned fragments, this shows that there is an additional active promoter immediately upstream of *repJ*. These results indicate that the REx-C module contains at least two functional promoters for the transcription of gene *repJ* and that the failure of fragments CG and DG to replicate is not due to a lack of transcription of *repJ*, suggesting an additional role for SaL 1 and 2.

### The structure of the repJ replicon is only partially conserved in *Pseudomonas*

**Figure 2:**
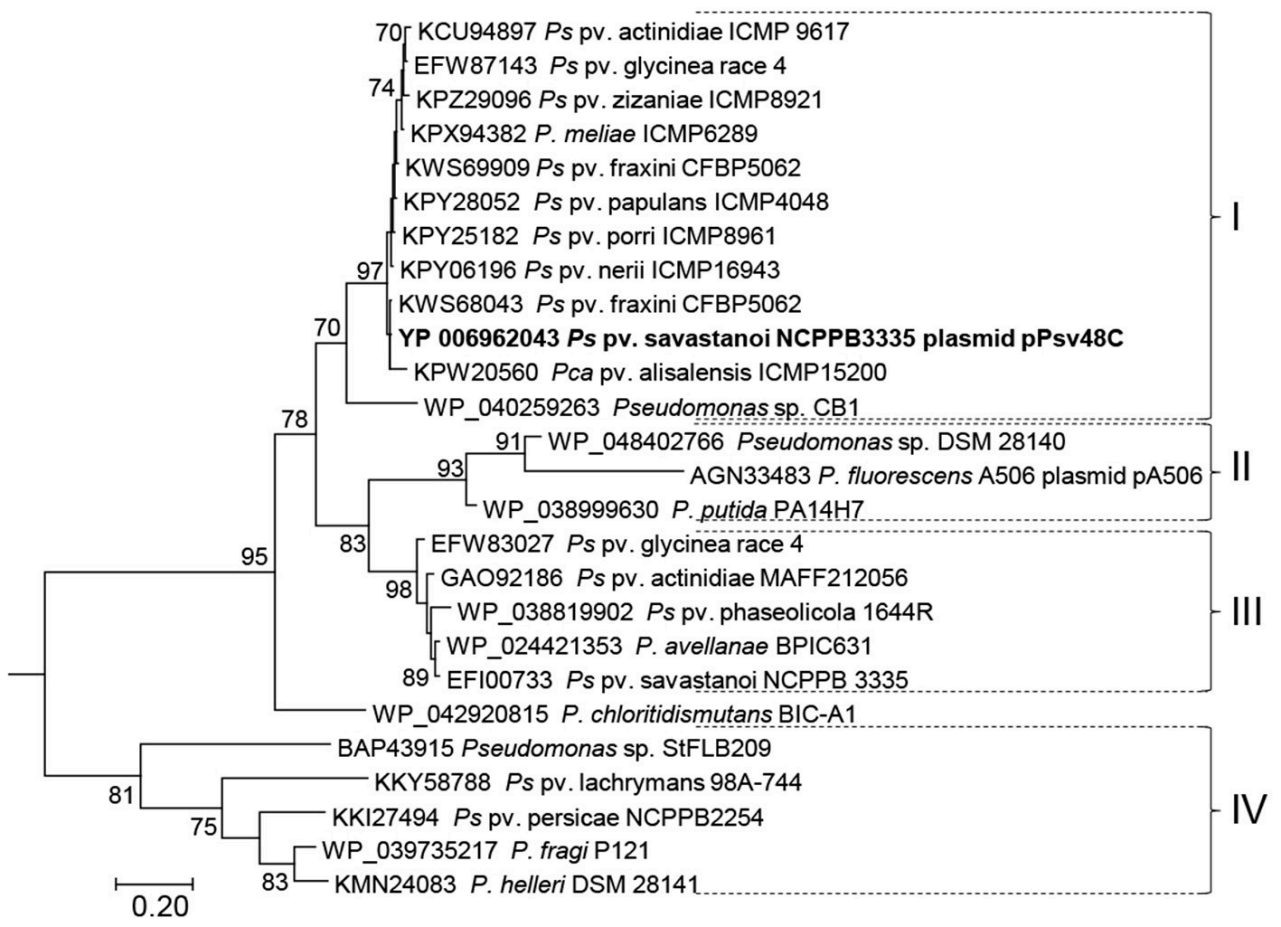
**Maximum likelihood phylogenetic tree of RepJ**. Protein sequences from species of *Pseudomonas* are indicated by their accession no. followed by strain designation; the first 39 positions of the alignment were discarded to eliminate biases due to differential annotation of the start sites. The tree was rooted with sequence WP_052264087.1 from *Azotobacter chrococcum* plasmid pAcX50f and bootstrap percentages of 500 replicates higher than 70% are shown close to each node. The tree is drawn to scale, with branch lengths measured in the number of substitutions per site. Brackets followed by roman numerals indicate clades discussed in the main text. Pca, *P. cannabina*; Pco, *P. coronafaciens*; Ps, *P. syringae*.

Blast comparisons revealed that a region of up to 2,785 nt containing the minimal RepJ replicon (coordinates 28,783–31,067 in FR820587) is syntenic, with very high identity, in diverse genomoespecies of the *P. syringae* group as well as, with less identity, in a few other pseudomonads (not shown). Most of the homologs are from draft genomes and it is not possible to clearly determine if they localize to the chromosome or to plasmid sequences. Sequence variation among the homologs from *P. syringae* was not distributed randomly (Figure S3): whereas the stems from SaL 1, 2, and 3 were identical in all sequences, there was a high sequence variation in the loop of SaL 2, in the 3′ end of gene *repJ* and in SaL 4 and 5, downstream of this gene. As it occurs with the *repA* gene (Gibbon et al., 1999), the nucleotide variation in *repJ* (Figure S3) leads to a higher degree of variation in the C-terminal end of the deduced product; the phylogeny of a selection of these products is shown as clade I in Figure 2.

Using blastp, we found RepJ homologs only within members of Pseudomonadales, mainly in species of *Pseudomonas*, and in a few *Desulfovibrio* spp. strains. An ML tree with selected sequences (Figure 2) grouped homologs in four well-defined clades; from this tree we can also infer that the RepJ replicon has recently moved horizontally among pathovars and species of the *P. syringae* complex and that certain strains contained two *repJ* homologs (e.g., *P syringae*. pv. fraxini CFBP5062 in clade I). The minimal RepJ replicon is moderately conserved and syntenic among homologs from clades I and II (Figure S4). Of note, the REx-C modules from the RepJ replicons from clade I were more similar among themselves than to the modules from RepA-PFP replicons, indicating that these RepJ replicons are long-time inhabitants of pseudomonads. Conversely, the REx-C module is not conserved among members in clades III and IV whereas the region downstream of *repJ* shows a degree of conservation (Figure S4); additionally these homologs show a genetic organization that is conserved among clade members, but different than that from members of clades I and II.

A 2,808 nt fragment (positions 35,079–37,886, accession no. KB644113) containing the *repJ* homolog from the chromosome of strain NCPPB 3335 (PSA3335_1080, clade III; see Figure S4) cloned in pKMAG did not generate any transformant after electroporation into *P. syringae* pv. syringae B728a or *P. syringae* pv. savastanoi UPN912, whereas in the same experiments we obtained hundreds of clones using the minimal RepJ replicon from pPsv48C. These results suggest that the chromosomal homolog from strain NCPPB 3335 is not able to sustain autonomous replication.

### The REx-C module associates to REx-R modules of diverse families and is exchanged among them

**Figure 3:**
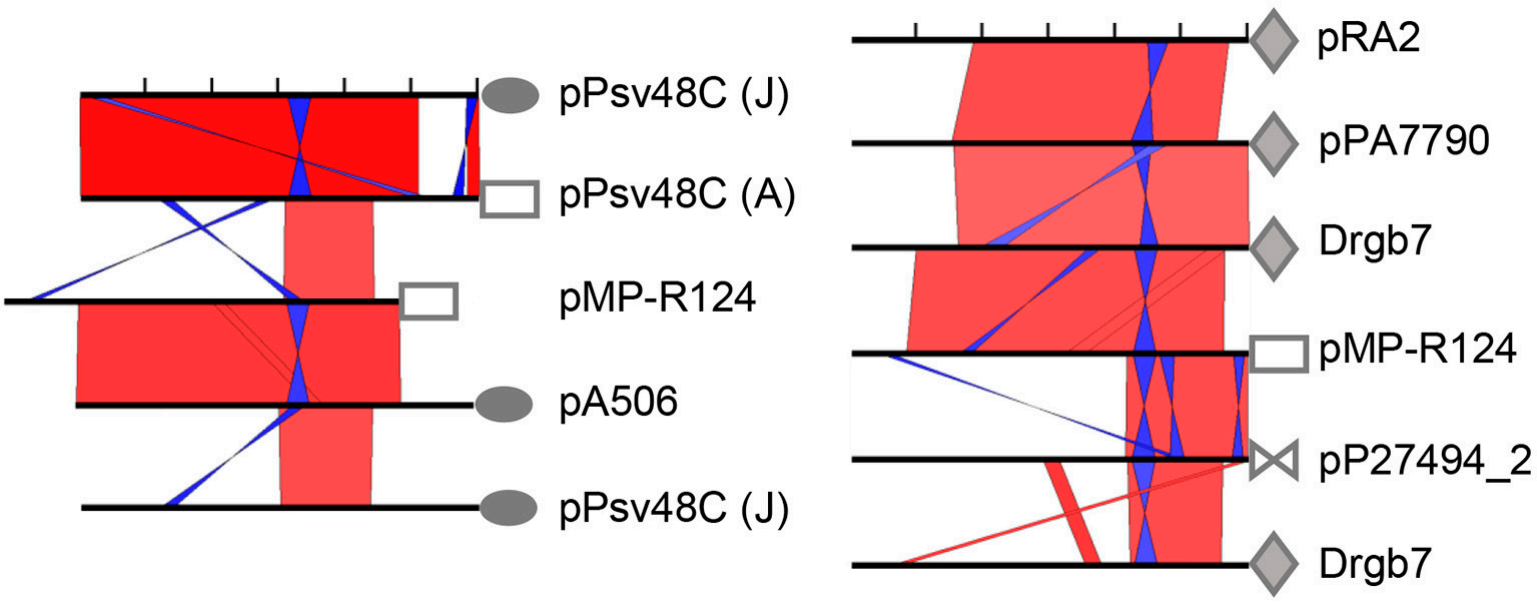
**The REx-C module is conserved among four non-homologous replicon families and subjected to recombination among them**. Graphical view of NCBI blastn comparisons of the 600 nt region preceding the start codon of replication initiator protein genes (black bars, ticks every 100 nt), displayed with the ACT software; default algorithm parameters were used, except for a word size of 7. Red and blue bars indicate collinear and inverted regions of identity, respectively, with color intensity proportional to identity; only matches larger than 15 nt are shown. Symbols are followed by their corresponding plasmid name and are identical for *rep* genes of the same homology family. Sequence accession no. are pA506, NC_021361; plasmid Drgb7, KT351738; pMP-R124, NZ_CM001562; pP27494_2, CP015602; pPA7790, CP015000; pPsv48C, FR820587; pRA2, U88088. A and J refer to *repA* and *repJ*, respectively, from pPsv48C.

Blastn comparisons showed that the REx-C module is present, with varying degrees of conservation, preceding the *rep* genes from at least four non-homologous replicon families from *Pseudomonas* (Figure 3; Table S1), whereas nucleotide identity is rapidly lost shortly after the CDS start codon. These families include the RepA-PFP family from *P. syringae* and other bacteria (exemplified by *repA* from pPsv48C) (Bardaji et al., 2011), the RepJ family (among others, *repJ* from pPsv48C and pA506) (Bardaji et al., 2011; Stockwell et al., 2013), and what we designated the RepA-RA2 family (named after pRA2) (Kato and Mizobuchi, 1994) and the RepA-Pa family (named here after the *P. antarctica* PAMC 27494 plasmid pP27494_2). The highest level of sequence conservation of the REx-C module among representatives of the four families is in a 175–194 nt fragment that spans SaL structures 1–3 (Figure S5). The stem sequences from SaL 1 (the putative transcriptional terminator for the antisense RNA), as well as the stretch of adenines in either side of them (Gibbon et al., 1999), are almost perfectly conserved and changes in one arm of the stems are usually compensated with complementary changes in the other arm (Figure S5) (Gibbon et al., 1999). The other structures are also well-conserved among the four replicon families; however, the number and position of palindromes, which could form SaL structures, are variable among replicons (Figure 3; Figure S5). Therefore, the stark conservation of the REx-C module suggests that it contains features universally essential for plasmid replication in species of *Pseudomonas*. Remarkably, we did not find REx-C sequences conserved in the control region of ColE2 replicons, which contain a replication initiator protein homologous to that from RepA-PFP replicons, or in any other plasmid outside of the genus *Pseudomonas*, indicating a diversity of REx-C modules among homologous theta replicons.

As described above, the REx-C modules accompanying genes *repA* and *repJ* from pPsv48C are nearly identical (Figure S1), although there is a gradient of conservation of this module along homologs of their corresponding gene families (Figure 3, Figures S3, S4) (Gibbon et al., 1999; Sesma et al., 2000; Stavrinides and Guttman, 2004). Additionally, the REx-C module preceding *repJ* from pA506 shows a very high degree of identity to that preceding *repA* from pMP-R124 (a PFP plasmid), whereas they are less similar to REx-C modules from replicons of their same family (Figure 3). These results show that there is frequent horizontal exchange of REx-C modules among RepA-PFP and RepJ replicons.

The results of multiple blast comparisons indicate that this exchange of REx-C modules also occurs with other replicon families. To illustrate this, we did a blast comparison against the non-redundant nucleotide collection (December 2016) of the 600 nt fragment immediately preceding the start codon for gene *repA* from the PFP plasmid pMP-R124, which comprises the REx-C module. The *rep* genes found immediately downstream of the first eight homologous sequences retrieved belong to the RepA-PFP, RepJ and RepA-RA2 families (Table S2). As before, and as an example, the REx-C module from pMP-R124 (RepA-PFP) shows a higher identity to pA506 (RepJ) than to the REx-C module from pPT14-32 (RepA-PFP) (Figure 3; Table S2). Incidentally, pMP-R124 and plasmids from the RepA-RA2 and RepA-Pa families also have a shorter REx-C module lacking the putative leader peptide gene sequence (Figure 3 and Figure S5). Together, these results indicate that the REx-C modules from these four families evolved vertically within the family, but were also subjected to horizontal exchange between members of other replicon families. Likewise, they indicate that plasmids from these four families have similar mechanisms of initiation of replication and its control.

### The REx-C module is functionally exchangeable among different replicons

**Figure 4:**
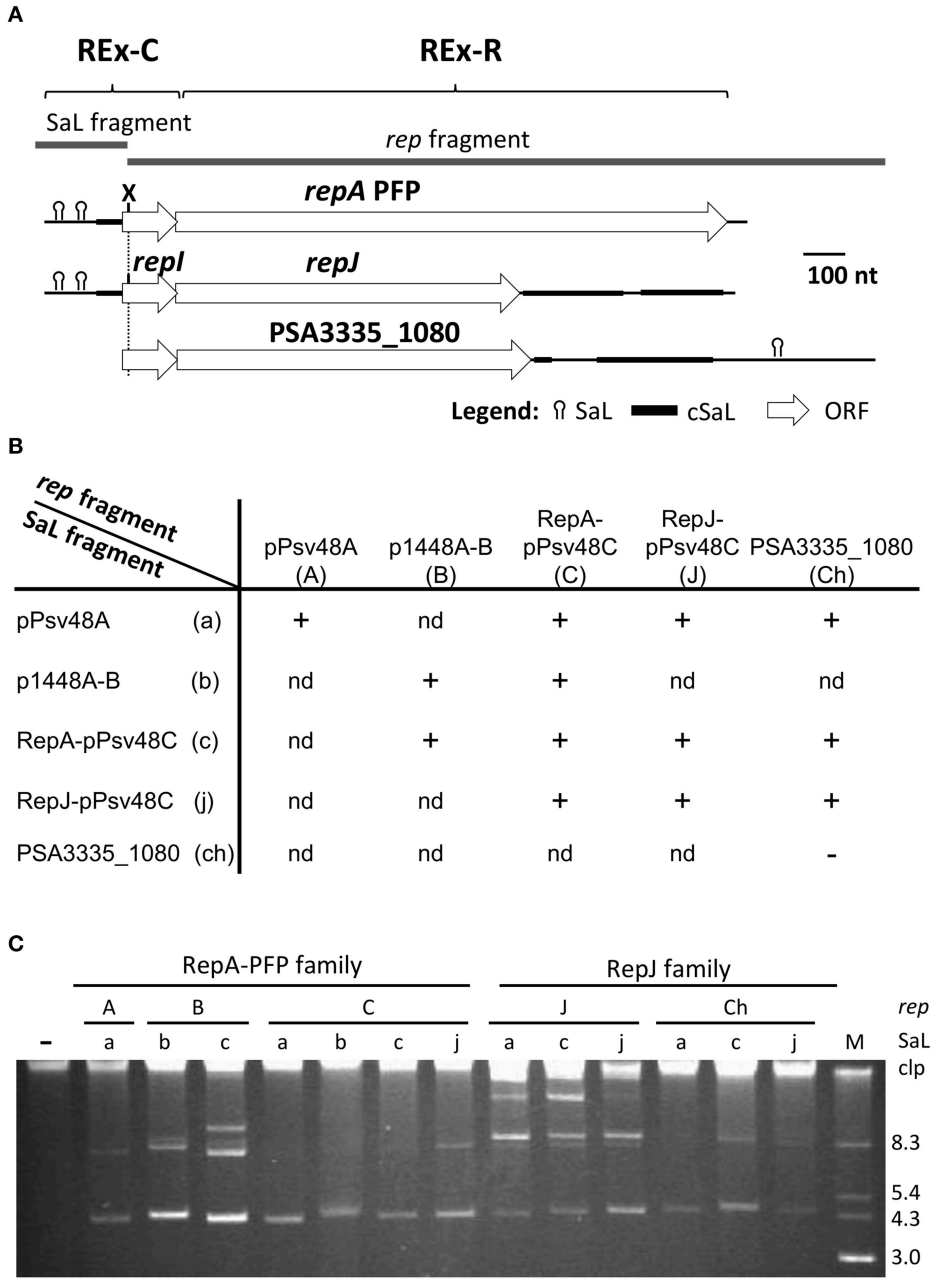
**The REx-C modules are functionally exchangeable within and among REx-R families. (A)** Schema of the DNA fragments exchanged for the construction of chimeras, which were cloned in vector pKMAG immediately after the transcriptional terminator. X, XmnI restriction site. **(B)** Autonomous replication in *P. syringae* pv. syringae B728a of wild type and chimeric DNA regions: +, autonomous replication; -, no replication; nd, not determined. **(C)** Undigested plasmid profile gels of B728a harboring the different wild type and chimeric clones, with numbers indicating the combination of SaL and *rep* fragments as in **(B)**; discrete plasmid bands evidence autonomous replication, with multiple bands being different topological forms and/or multimers. Lane (-) is the wild type strain B728a, plasmidless; lane M, size markers, in kb; Clp, chromosome and high-molecular-weight plasmid multimers.

Our comparative analyses of extant sequences (Figure 3) suggest that REx-C modules are freely exchanged among different replicons. However, previous works postulated that compatibility of coexisting RepA-PFP replicons was due to specificity between the C-terminal part of RepA and the loop sequence of SaL 2 (Figures S3, S6), which are highly variable and could coevolve for complementarity (Murillo and Keen, 1994; Gibbon et al., 1999; Stavrinides and Guttman, 2004; Ma et al., 2007). Additionally, the same pattern of variation is seen in a comparison of RepJ replicons (Figure S3). We therefore tested this putative specificity by swapping the respective SaL structures from the REx-C module (SaL fragment in Figure 4) and the *rep* fragments (partial *repI* plus REx-R, see Figure 4) from plasmids p1448A-B (RepA-PFP) and pPsv48C (RepA-PFP and RepJ) (Figure 4 and Figure S6). These two RepA-PFP initiator proteins are 88% identical (92% similar; Table S1 and Figure S6), while they are not homologous to RepJ (Table S1). Likewise, the three replicons show a different loop in SaL 2 and 1–3 nt changes within SaL 3 (Figure S6). In spite of the differences in sequence, all the analyzed chimeras were able to sustain autonomous replication in the plasmidless strain *P. syringae* pv. syringae B728a (Figure 4), indicating a lack of specificity between the SaL structures from the REx-C module and the rest of the replicon.

To test whether or not sequence variations in SaL 1 (Figures S1, S6) would be specific for each Rep protein, we evaluated chimeric clones containing the SaL fragment from plasmid pPsv48A and the *rep* fragments of the RepA-PFP or RepJ replicons from pPsv48C. These two RepA-PFP proteins, from pPsv48A and pPsv48C, are 97% identical (Table S1 and Figure S6), but the pPsv48A REx-C module (and consequently the SaL fragment tested) is comparatively shorter and with several changes in SaL 1 and 2. In spite of the differences, both chimeras replicated autonomously in strain B728a (Figure 4), indicating that sequence variations in the loop sequence from SaL 1, and in the sequence upstream of this structure, do not significantly impact replication ability.

Finally, none of the four SaL fragments cloned in pKMAG generated any transformants when transferred to strains B728a or 1448A (the latter containing two plasmids that could supply the RepA initiator *in trans*), suggesting that the REx-C module cannot sustain replication by itself.

### The REx-C module confers replication ability to a non-replicative *repJ* homolog

The REx-C module showed no apparent specificity with the associated Rep protein and is readily exchanged among replicons (Figures 3, 4), serving as a portable putative replication control region. Therefore, it is feasible that REx-C modules could move within bacterial genomes, co-opt genes with the appropriate characteristics and thereby directly create new autonomous replicons. To broadly test this, we examined the effect of the SaL structures from the RepA-PFP and RepJ replicons from pPsv48C on the replication ability of gene PSA3335_1080. This gene is located in the chromosome of *P. syringae* pv. savastanoi NCPPB 3335 and its deduced product shows 72.7% identity (81.2% similarity) with that from *repJ* (Figure 2 and Figure S6; Table S1), but does not replicate autonomously (see above).

We therefore amplified and cloned a 1,772 nt fragment containing gene PSA3335_1080 plus the upstream 114 nt, to preserve the RBS and a similar spacing to the SaL structures as with *repJ*, and 815 nt downstream, spanning sequences homologous to SaL 5 (Figure 4). This fragment was ligated in the proper orientation to a partial fragment of the REx-C module containing the SaL structures preceding either *repA* or *repJ* from pPsv48C (279 nt), or preceding *repA* from pPsv48A (203 nt). The three constructions replicated in *P. syringae* pv. syringae B728a with very high efficiency (Figure 4). Disruption of the PSA3335_1080 reading frame by filling-in an internal restriction site abolished the replication ability of the clone containing the SaL fragment from the RepA-PFP replicon of pPsv48C. These results indicate that acquisition of a REx-C module can immediately confer autonomous replication ability to sequences containing PSA3335_1080, and that replication is dependent on the activity of this gene.

### Replicon incompatibility associates to the REx-C module

Their frequent exchange (Figure 3), and our experiments with chimeras (Figure 4), indicate that the REx-C modules have a low, or no specificity for their cognate REx-R module and that they probably confer a strong selective advantage. Among other possibilities, we speculated that the exchange of these modules could be a way to reduce or evade incompatibility, especially for coexisting plasmids carrying related replicons, such as the PFP group. The ColE2 replicon, related to the RepA-PFP replicons (Gibbon et al., 1999), contains two incompatibility determinants, corresponding to the control region and to the origin of replication (Tajima et al., 1988; Hiraga et al., 1994). Importantly, the RepA-PFP replicons contain sequences highly similar to the origin of replication of ColE2 replicons (Figure S7) (Yagura et al., 2006), including the primer RNA sequence (AGA), and located either within the RepA coding sequences or, for pMP-R124, situated some 300 nt after the *rep* gene stop codon.

**Figure 5:**
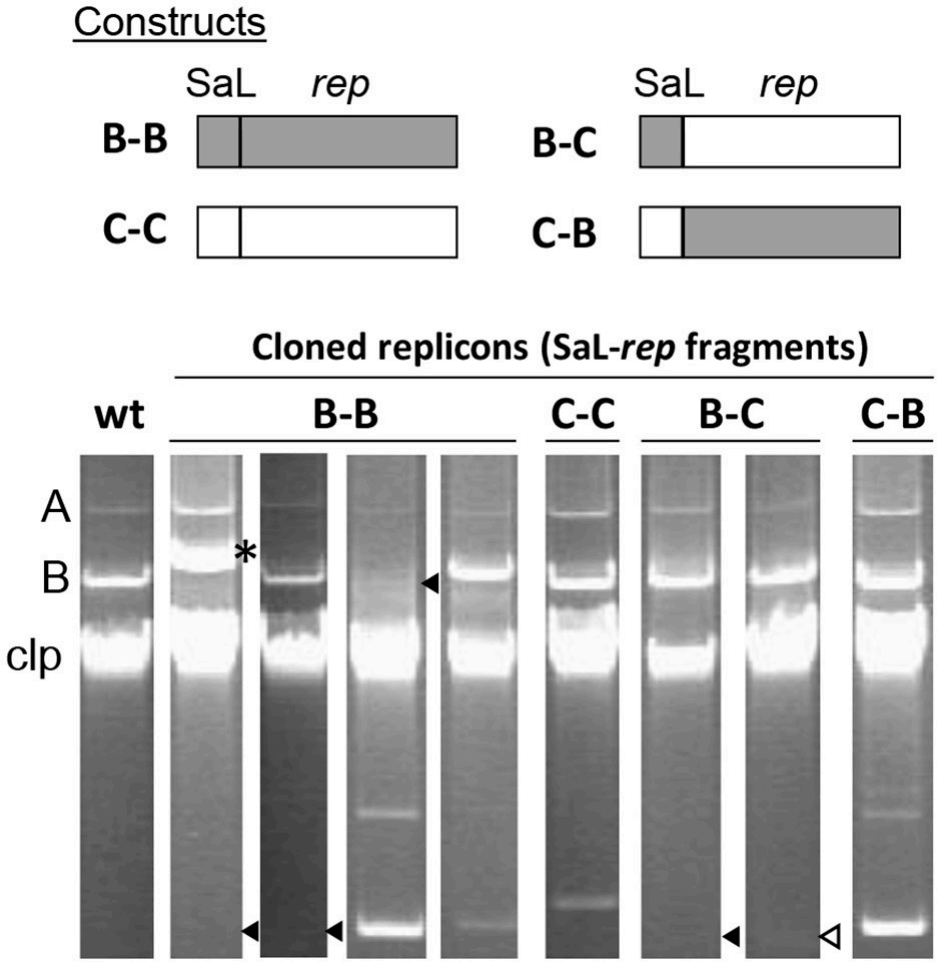
**The REx-C module, but not the REx-R module, confers plasmid incompatibility**. The cartoon represents the native replicons and chimeras of the SaL fragment from the REx-C module and the *rep* fragments of plasmids p1448A-B (B) and pPsv48C (C), as indicated by the lettering, cloned in pKMAG. The bottom panels show representative examples of plasmid profiles of strain *P. syringae* pv. phaseolicola 1448A (wt) and transformants containing the native replicons and chimeras. Strain 1448A contains naturally the native RepA-PFP plasmids p1448A-A (A, 132 kb) and p1448A-B (B, 52 kb). Incompatibility is deduced from the loss of p1448A-B, or for the inability of the incoming plasmid to be maintained as an independent molecule. The asterisk indicates cointegrated plasmids; black triangles, missing plasmid band; open triangle, faint plasmid band (visible in the actual gel, but not in the picture). Clp, chromosome and linearized plasmids.

We thus evaluated possible changes in the incompatibility behavior of chimeric replicons by transforming strain *P. syringae* pv. phaseolicola 1448A with the native RepA-PFP replicons from plasmids p1448A-B (B) and pPsv48C (C), and with their two corresponding chimeras of SaL and *rep* fragments (combinations B-C and C-B of the SaL-*rep* fragments; see Figures 4, 5). Strain 1448A contains naturally the native RepA-PFP plasmids p1448A-A (132 kb) and p1448A-B (52 kb). As expected, the native replicon from pPsv48C did not show any obvious incompatibility, producing a large number of transformants and coexisting with the two native plasmids from strain 1448A (Figure 5, lane C–C). The same results were observed with the C–B chimera (Figure 5, lane C–B), containing the SaL structures from plasmid C and the *rep* fragment from plasmid B. Conversely, the native replicon from plasmid B (B–B in Figure 5) and the B–C chimera generated about half the number of transformants than the two other replicons tested, taking double the time to reach colonies of the same size. Incompatibility mediated by the native replicon from plasmid B and the B-C chimera was evident in plasmid profile gels, where they either cointegrated with p1448A-B or induced its loss, or appeared with an apparently reduced copy number (Figure 5). These results indicate that strong replicon incompatibility between RepA-PFP replicons is associated to the REx-C module and, unlike what happens with ColE2 replicons (Tajima et al., 1988; Yasueda et al., 1994), not to the Rep protein or the origin of replication and that it can be overcome by only a few nucleotide changes in this module.

## Discussion

Modularity, which can be broadly defined as the degree to which a system is made up of relatively independent but interlocking parts, is ubiquitous in biology at all organization levels (Wagner et al., 2007; Kreimer et al., 2008). In this work, we demonstrate that diverse plasmid replicons from *Pseudomonas* are also modular, being composed of discrete functional units, or modules, that are readily and frequently exchanged among unrelated systems, colliding with the traditional view of minimal replicons as heritable units that evolve as a whole and that can be classified in more or less coherent incompatibility and phylogenetic groups (del Solar et al., 1998; Petersen, 2011).

We have found extensive evidence of modularity in plasmids from many diverse species of *Pseudomonas*, including environmental species as well as plant, animal and human pathogens of paramount significance, such as *P. syringae* and *P. aeruginosa* (Figures 2, 3, Table S2, and not shown). Nevertheless, it is highly likely that this concept is also applicable to plasmid replicons consisting of *rep* gene and control systems from other organisms. In particular, homologous and/or site-specific recombination has been postulated to contribute to a similar exchange of control and *rep* modules in related enterobacterial plasmids of the IncIα and IncFII incompatibility groups (Kato and Mizobuchi, 1994) of the class A theta replicons, as well as different segments of ColE2 replicons (Hiraga et al., 1994); nevertheless, the concept of modularity as a general organizational model for replicons did not emerge from these studies. Based on their properties as functionally independent and exchangeable units, we defined two *r*eplicon *ex*changeable modules and designated them as REx-C (*c*ontrol) and REx-R (*r*eplication) modules, respectively (Figure 1).

Module REx-C consists of the replication control region, and has variable size and configuration. As defined for the RepJ replicon from pPsv48C, REx-C is a small sequence that contains three potential SaL structures, a putative leader peptide, a putative antisense RNA, and the signals for transcription and translation of the *rep* gene (Figure S1). Based on these characteristics, and by structural similarity to previously described replicons, it is highly likely that the REx-C module is involved in copy number control by the antisense RNA (del Solar et al., 1998; Brantl, 2014). Sequence comparison of extant replicons (Figure 3) and functional assays (Figures 1, 4) indicate that this module is an autonomous but essential part of the replicon, and that it is functionally exchangeable among four non-homologous REx-R families (Table S1). The module contains a core region of 175–194 nt highly conserved among members of the four families (Figure 3, Figure S5) and that spans the three SaL structures essential for autonomous replication of the minimal RepJ (Figure 1) and the RepA-PFP replicons (Murillo and Keen, 1994; Gibbon et al., 1999). We showed that chimeric replicons containing different combinations of SaL structures and *rep* genes from the RepA-PFP and RepJ families were fully functional (Figure 4), demonstrating the independent functionality of the REx-C module and a lack of specificity with the REx-R module. Importantly, the *rep* fragments used for the construction of chimeras retained their corresponding peptide leader gene sequence and the RBS for the *rep* gene (Figure 4), suggesting that control of replication by the REx-C module does not involve the formation of secondary structures (pseudoknot) with sequences surrounding the *rep* gene. Additionally, the SaL structures also conferred replication ability to the *rep* fragment containing PSA3335_1080 (Figure 4), whose sequence is poorly conserved compared with the other replicons used for the construction of chimeras. Work with pA506 suggested that clones containing its REx-C module, without the *repJ* gene, were able to sustain autonomous replication (Stockwell et al., 2013), although these authors did not conclusively eliminate the possibility of ectopic integration. Conversely, evidence presented here and elsewhere (Murillo and Keen, 1994; Kwong et al., 1998; Gibbon et al., 1999; Sundin et al., 2004) shows that diverse REx-C modules were unable to replicate autonomously. Consequently, their functionality and conservation suggest that SaL structures in the REx-C module are essential for replication and replication control of a wide variety of replicons. This likely happens because the REx-C module harbors the signals required for expression of the *rep* gene, whose accessibility and proper recognition would be modulated by the interaction between the antisense RNA and the mRNA. Since the antisense RNA and its target sequence on the *rep* mRNA would be coded for in complementary strands of the same SaL region (Figure S1), they always show a perfect complementarity independently of any other downstream sequence. Therefore, the REx-C module would then act as an independent, self-contained portable unit for the control of replication.

The SaL structures are followed by a putative leader peptide that is present in diverse replicons as part of the region for the control of plasmid copy number (del Solar et al., 1998; Brantl, 2014). Previous works (Wagner et al., 1987; Blomberg et al., 1992) also showed that the level of expression of the *rep* gene is controlled by the level of translation of the leader peptide gene, and not by its product. This is compatible with the fact that an in-frame deletion of most of the putative leader peptide gene (*repI*) sequence in the RepJ replicon did not have any significant effect on replication (Figure 1). Likewise, lack of function for the product of this gene could justify the large sequence differences in the leader peptide genes of the RepJ and RepA-PFP replicons from pPsv48C, despite the rest of the REx-C module being highly similar (Figure S1). Remarkably, some of the REx-C modules examined here (e.g., from pMP-R124 and pRA2) lack the leader peptide gene, with the consequence that the start of the *rep* gene is in very close proximity to the end of SaL 3 (Figure 3 and Figure S5). Nevertheless, the REx-C module is highly flexible and can accommodate different structures to ensure functionality in a diversity of replicon arrangements. For instance, functionality of the pRA2 replicon required a large region upstream of the *rep* gene, containing in this order: four potential iterons, an additional small gene (*repB*, 240 nt), and a fragment containing the conserved SaL structures shown in Figure S5 (Kwong et al., 1998).

We found four different families of REx-R modules defined by groups of homology of the corresponding Rep proteins (Figure 3 and Table S1). The family RepA-PFP is composed of a single gene, coding for a large protein of as much as 437 amino acids homologous to many replication proteins from diverse species of Gram-negative and Gram-positive bacteria, including ColE2 replicons (Gibbon et al., 1999; Yagura et al., 2006). The RepA-PFP proteins contain highly conserved primase, PriCT and HTH domains, with the vegetative origin of replication located in the 3′ end of the *rep* gene or immediately after this gene (Figure S7) (Yagura et al., 2006). The other REx-R families contain smaller *rep* genes, with deduced products ranging from 269 to 341 amino acids and lacking homology to protein families and domains included in the InterPro database (Mitchell et al., 2015). These genes might also be accompanied by downstream sequences that can form complex folding structures and that are essential for replication, as it is the case with the RepJ replicon (Figure 1) and pRA2 (Kwong et al., 1998). Directed mutagenesis of the *repJ* (Figure 1), PSA3335_1080 and the *repA*-PFP (Gibbon et al., 1999) *rep* genes, demonstrates that they are essential for replication. Thus, the diversity of the REx-R modules found here again stresses the functional universality of the REx-C module.

The exchange of REx modules among disparate replicons is intriguing and can confer diverse evolutionary advantages, such as increasing their competitiveness by acquiring modules that are better adapted to their particular bacterial host. In this respect, the type of REx-C module characterized here is probably specific to *Pseudomonas* and its general presence in diverse replicons likely facilitates plasmid survival in these bacteria. Indeed, we were unable to find sequences homologous to the REx-C module in any other bacteria outside the genus *Pseudomonas*. Additionally, the RepA-PFP replicons analyzed here contain specific REx-C modules that are different from those associated to homologous RepA proteins, such as those from the enterobacterial ColE2 plasmids. Another likely advantage of modularity is to favor the coexistence of highly related replicons. This type of coexistence has been documented for PFP plasmids of pseudomonads (Murillo and Keen, 1994; Sundin, 2007) and *repABC*-type plasmids from rhizobia (Cevallos et al., 2002), and it also likely happens with RepJ replicons (Figure 2). The analysis of chimeric replicons indicates that plasmid incompatibility is conferred by the REx-C module (Figure 5), in agreement with previous results indicating a partial incompatibility of the corresponding module from plasmid pPT23A (Gibbon et al., 1999). Additionally, the incompatibility between plasmid p1448A-B and its cloned replicon was bypassed by swapping the REx-C module of the cloned replicon with another one differing in the sequence of the loop from SaL 2, as well as in a few other nt positions (Figure 5 and Figure S6). Therefore, the exchange of REx-C modules could be a rapid and efficient way of reducing or eliminating incompatibility among highly related PFP plasmids, which usually carry genes conferring important adaptive advantages (Vivian et al., 2001; Sundin, 2007). Additionally, it is also likely that the coexistence of related modular replicons, requiring similar cellular resources, could help to streamline the replication process and reduce the metabolic burden of plasmids.

A further and exciting consequence derived from the modularity of replicons, is that their combination could potentially produce new replicons. In particular, the independent functionality of the REx-C module could allow it to co-opt appropriate genes that might then function as Rep initiators and result in new autonomously replicating molecules. But, would the bacterial gene pool contain genes that will function as plasmid replication initiators when associated to a REx-C module? Our experiments indicate that this is indeed the case: the non-replicative chromosomal *repJ* homolog PSA3335_1080 could replicate autonomously when preceded by a DNA fragment containing the SaL structures from the REx-C module. PSA3335_1080 is a chromosomal gene widely distributed in *P. syringae* and related species, conserving synteny with its adjacent sequences, which suggests that is a long time inhabitant of these bacteria possibly having a functional role different from replication. A priori, there could be a variety of genes that could generate new replicons when combined with an appropriate REx-C module and their identification could be challenging. For instance, manual or automatic prediction of structure or function of the RepJ protein and homologs would likely be unsuccessful because they lack obviously conserved domains typical of Rep proteins, and this might also be the case for other genes that could be recruited as replication initiators. Therefore, and as occurs with other systems (Agapakis and Silver, 2009; Lorenz et al., 2011; Melo et al., 2016), modularity of origins of replication will undoubtedly favor their evolution and adaptability, for instance by reducing incompatibility and metabolic load to the bacterial host, but could also be facilitating the generation of new plasmid replicons, with the concomitant possibility of immediate mobilization of associated chromosomal genes.

## Materials and methods

### Bacterial strains, plasmids, and growth conditions

Bacterial strains and plasmids used in this work are detailed in Table S3. *E. coli* strains DH10B and GM2929 (*dcm*^−^, *dam*^−^), when unmethylated DNA was needed, were used for DNA manipulations and were grown in LB at 37°C. Strains *P. syringae* pv. phaseolicola 1448A (Joardar et al., 2005), pv. syringae B728a (Feil et al., 2005), and pv. savastanoi NCPPB 3335 (Rodríguez-Palenzuela et al., 2010) and UPN912, which derives from strain NCPPB 3335 and is cured of its three native plasmids (M. Añorga, unpublished results), were propagated using King's medium B (King et al., 1954) at 25°C. When necessary, media were supplemented with 100 μg ml^−1^ ampicillin or 25 μg ml^−1^ kanamycin. We used a mixture of plasmids pME6031 (8.3 kb), pBBR1MCS-2 (5.4 kb), pKMAG-C (4.3 kb) and pBlueScript II (3.0 kb) (Table S3), purified from *E. coli* using the Illustra plasmidPrep Mini Spin kit (GE Healthcare, UK), as size markers in plasmid profile gels.

### Molecular techniques

DNA was amplified using a high fidelity enzyme (PrimeStar HS, Takara Bio Inc., Japan) and cloned using the CloneJET PCR Cloning Kit (Thermo Scientific) or the pGEM-T Easy Vector System (Promega), following the manufacturer's instructions. For plasmid profile gels, DNA was purified by alkaline lysis and separated by electrophoresis in 0.8% agarose gels with 1xTAE as described (Murillo et al., 1994). Plasmids were transferred to *P. syringae* by electroporation (Choi et al., 2006). For RT-PCR analysis, DNA-free RNA was obtained from bacterial cultures grown overnight in medium B using TriPure Isolation Reagent (Roche Diagnostics) and Ambion TURBO DNA-free Kit (Life Technologies). Concentration and purity of RNA were determined spectrophotometrically, and its integrity confirmed by electrophoresis in agarose gels. cDNA was synthesized from RNA using the ImProm-II reverse transcriptase system (Promega), following the manufacturer's recommendations. Primer Int_R, 5′-GCCGGTGCAGAGATACCC-3′, specific for the sense transcript of *repJ*, was used for the reaction: at 25°C for 5 min for primer annealing, 60 min at 42°C for reverse transcription, and 15 min at 70°C for enzyme inactivation. cDNA was amplified using primers B (5′-CGATGTAGATTCACGAATCGCAG-3′), C (5′-CTGATTATGGCGTTCACTGC-3′), or D (5′-TGCAAGCTGTCTAAAGTGAAGC-3′) together with 1R (5′-GCTGTTGTTCAGAGAGATGACG-3′), to determine the size of the transcript, or primer pair Int_F (5′-GAGAAGTTTCTGGCCATCGAG-3′) and Int_R (5′-GCCGGTGCAGAGATACCC-3′) to analyze the transcriptional activity of gene *repJ* (see Figure 1). The program used comprised 30 cycles (94°C for 30 s, 58°C for 30 s, and 72°C for 30 s) plus a final extension step of 6 min at 72°C. Control reactions included PCR amplification of pure extracted RNA, to verify the absence of contaminating DNA; amplification of purified DNA, to verify the reaction conditions, and amplification of an internal fragment of gene *gyrB*, to confirm the synthesis of cDNA.

### Bioinformatics

Multiple-sequence alignments using Muscle, determination of the optimal substitution model, and Maximum likelihood phylogenetic tree construction using the JTT matrix-based model with a gamma distribution with 5 categories were done using MEGA7 (Kumar et al., 2016); confidence levels of the branching points were determined using 500 bootstraps replicates. Searches for sequence similarity in the NCBI databases were done using the BLAST algorithms (Hubbard et al., 2008) and sequences were aligned on-line using the MULTALIN program (Corpet, 1988) or the tools in the EMBL-EBI server (http://www.ebi.ac.uk/Tools/msa/). Search for protein motifs and fold recognition was done using the InterPro (Mitchell et al., 2015) (http://www.ebi.ac.uk/interpro/) and the Phyre2 (Kelley et al., 2015) web servers. Genome and nucleotide sequences were visualized and manipulated using the Artemis genome browser; when necessary, blast comparisons were visualized with ACT (Carver et al., 2008). Oligonucleotide primers were designed using Primer3plus software (Untergasser et al., 2012). Promoters were predicted using the online BPROM server (http://www.softberry.com). DNA or RNA Folding predictions were done using the Mfold web server (Zuker, 2003) using the default settings, except for a folding temperature of 25°C. Patterns of nucleotide polymorphism were calculated using DnaSP v 5.10.01 (Librado and Rozas, 2009).

### Construction of vectors

The *E. coli* pKMAG vector and the *E. coli*-*Pseudomonas* pKMAG-C vector were constructed to examine, respectively, the replication ability of cloned fragments and the expression of *repJ* in these fragments. To construct pKMAG, a PCR-amplified fragment from the pK184 vector (positions 71-2,064; accession no. U00800) (Jobling and Holmes, 1990), retaining the kanamycin resistant gene and the origin of replication and lacking the *lacZ* promoter, and a fragment from pME6041 (Heeb et al., 2000) (positions 3,809–4,255; accession no. AF118812), containing its polylinker and the T4 transcription terminator, were ligated together resulting in the new vector. The RepA-PFP replicon from pPsv48C (positions 41,791–1,428; accession no. FR820587) was then cloned into the unique AscI site of pKMAG, resulting into pKMAG-C. Construction details are included in Figure S2.

### Replication assays

Fragments used for the definition of a minimal RepJ replicon were amplified by PCR from strain Psv48ΔAB (Bardaji et al., 2011), a derivative of strain NCPPB 3335 containing only pPsv48C, cloned into pJET1.2 (Thermo Scientific) and then subcloned into pK184 in both directions, pKMAG and/or pKMAG-C. Primers used for amplifications were A (5′-AAAGCAGCGGATTTTGTAGG-3′), B, C, and D (described above), as forward, and E (5′-GACGCTAGGAGCCTATCCAG-3′), F (5′-TCCCTGTTTTTCCTGAAAGG-3′), and G (5′-GGTCGAACCGACCAACTG-3′), as reverse primers (see Figure 1 and Figure S1). For the construction of chimeric replicons we first cloned in pKMAG amplicons containing the RepA-PFP and RepJ replicons, including the REx-C module in a 390–391 nt upstream fragment, from plasmids pPsv48C (coordinates 41,714–1,336, for RepA-PFP, and 23,309–31,027, for RepJ, from accession no. FR820587) and p1448A-B (coordinates 51,624–1,650 from accession no. CP000060). The SaL (coordinates 78,056–78,262, from accession no. FR820585) and the *rep* (coordinates 78,254–1,360, from FR820585) fragments from pPsv48A were amplified by PCR and cloned separately in pJET1.2 to reconstruct an XmnI site present in many RepA-PFP replicons and missing from pPsv48A, resulting in a T
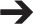
C change in pos. 78,263. A double digestion XmnI-EcoRI or XmnI-XhoI of the resulting clones liberated the *rep* genes (*rep* fragment), leaving a 203–280 nt fragment attached to pKMAG that contained SaL structures 1 to 3 from the REx-C module (SaL fragment). The SaL and *rep* fragments were then separated by electrophoresis, purified and ligated in the appropriate combinations for the construction of chimeras. A 2,808 nt amplicon containing PSA3335_1080 (35,079–37,886 from accession no. KB644113), including 1,150 nt upstream and 815 nt downstream of the CDS, was cloned in pKMAG to evaluate replication of this gene in its native configuration. Additionally, an amplicon similar to the previous one but lacking the first 1,036 nt (36,115–37,886 from accession no. KB644113), and with an A
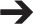
T change in pos. 36,115, was ligated in the proper orientation to the appropriate XmnI-EcoRI clones in pKMAG generated above and containing the SaL fragments from *repA* or *repJ* from pPsv48C, or from pPsv48A. For functional analyses of replication initiation protein genes, replicative constructions containing *repJ* and PSA3335_1080 were digested using a unique restriction site internal to each CDS, filled-in with Klenow enzyme (New England BioLabs Inc., UK) and subsequently religated. Enzymes used for disrupting the CDSs by changing the reading frame were SexAI, adding 5 nt to the *repJ* CDS, and EcoNI, adding 1 nt to the CDS of PSA3335_1080. For all replication experiments, at least two amplicons from two separate amplification experiments were cloned and tested; the identity and integrity of all clones was confirmed by sequencing. Replication ability was assessed by electroporation into the plasmidless strains *P. syringae* pv. syringae B728a and UPN912. Plasmids were confirmed to replicate autonomously by their ability to generate antibiotic-resistant transformants and, necessarily, by their appearance as independent bands in undigested plasmid profile gels, basically as described (Murillo and Keen, 1994; Sesma et al., 1998). Experiments were repeated at least three times, with similar results.

### Incompatibility assays

Incompatibility between native plasmids and cloned replicons was analyzed essentially as described (Nordström, 1993; Sesma et al., 1998). Briefly, constructs in pKMAG containing the native RepA-PFP replicons from plasmids pPsv48C (strain *P. syringae* pv. savastanoi NCPPB 3335) and p1448A-B (strain *P. syringae* pv. phaseolicola 1448A) and the corresponding chimeras of SaL and *rep* fragments, were isolated from *E. coli* and individually transferred to *P. syringae* pv. phaseolicola 1448A, which naturally contains the native plasmids p1448A-A (132 kb) and p1448A-B (52 kb) (Joardar et al., 2005). Resulting km^R^ transformants of strain 1448A were cultured overnight in liquid B medium with kanamycin at 25°C with shaking, and their plasmid content visualized by electrophoresis in plasmid profile gels to determine the possible eviction of native plasmids.

## Accession numbers

Sequences of vectors pKMAG and pKMAG-C are deposited in GenBank under accession numbers KX714576 and KX714577.

## Author contributions

LB and JM conceived the study and designed the experiments; LB and MA performed the experiments; LB, MA, JARM, GdS, and JM analyzed the data and interpreted the results; LB and JM drafted the manuscript with contributions from JARM and GdS; all authors read and approved the final manuscript.

## Funding

This work was funded by the Spanish Plan Nacional I+D+i grant AGL2014-53242-C2-2-R, from the Ministerio de Economía y Competitividad (MINECO), co-financed by the Fondo Europeo de Desarrollo Regional (FEDER). M.A. was supported by an FPI fellowship (reference BES-2012-054016, Ministerio de Ciencia e Innovación/Ministerio de Economía y Competitividad, Spain). The funders had no role in study design, data collection and interpretation, or the decision to submit the work for publication.

### Conflict of interest statement

The authors declare that the research was conducted in the absence of any commercial or financial relationships that could be construed as a potential conflict of interest.
