# Supplementary material for: Plasmid Replicons from Pseudomonas Are Natural Chimeras of Functional, Exchangeable Modules
Source: Front Microbiol. 2017 Feb 13;8:190. doi: 10.3389/fmicb.2017.00190 (PMC5304414; doi:10.3389/fmicb.2017.00190)
Supplement: Supplementary file 1 [file Table1.pdf]

Table S1. Amino acid identity among plasmid replication initiator proteins from four different replicon families[a]

Replicon family	RepA-PFP				RepJ			RepA-RA2				RepA-Pa
Accession number	CBZ40037	CBZ39919	AAZ38092	EJZ60922	CBZ40073	CP003042[c]	EFI00733	AAB70929	AP014863[b]	ALG88764	AIT41777	ANF89329
Plasmid	pPsv48C (RepA)	pPsv48A	p1448_B	pMP-R124	pPsv48C (RepJ)	pA506	PSA3335_1080	pRA2	pKF707	Drgb7	pLM8-P2	pP27494_2
pPsv48C (RepA)	100.0	96.5	88.1	65.0	20.1	18.8	19.5	16.3	12.9	16.8	15.48	16.9
pPsv48A	96.5	100.0	88.9	65.1	20.5	19.1	19.9	15.7	12.7	16.7	15.06	16.9
p1448A-B	88.0	88.9	100.0	65.7	20.5	17.8	20.3	13.4	13.3	15.4	13.39	17.3
pMP-R124	65.0	65.1	65.7	100.0	16.5	15.5	15.3	12.0	9.3	14.7	11.84	15.8
pPsv48C (RepJ)	20.1	20.5	20.5	16.5	100.0	65.9	72.7	23.8	19.8	19.2	21.74	14.7
pA506	18.8	19.1	17.8	15.5	65.9	100.0	63.3	25.6	20.8	20.9	23.20	15.3
PSA3335_1080	19.5	19.9	20.3	15.3	72.7	63.3	100.0	24.2	20.8	17.7	20.63	15.0
pRA2	16.3	15.7	13.4	12.0	23.8	25.6	24.2	100.0	58.3	65.6	67.29	22.2
pKF707	12.9	12.7	13.3	9.3	19.8	20.8	20.8	58.3	100.0	51.8	56.51	21.2
Drgb7	16.8	16.7	15.4	14.7	19.2	20.9	17.7	65.6	51.8	100.0	60.97	22.0
pLM8-P2	15.5	15.1	13.4	11.8	21.7	23.2	20.6	67.3	56.5	61.0	100.00	22.0
pP27494_2	16.9	16.9	17.3	15.8	14.7	15.3	15.0	22.2	21.2	22.0	21.97	100.0

[a] Figures represent percentage of amino acid identity in pairwise comparisons. The percent identity matrix was obtained from a multiple sequence alignment done with T-Coffee in the EMBL-EBI web server (http://www.ebi.ac.uk/Tools/msa/tcoffee/).

[b] The corresponding gene is not annotated in the sequence of plasmid pKF707; therefore, we compared the product deduced from positions 43,929-43,093 of sequence accession no. AP014863.

[c] The annotation of the deduced product of *repA* from pA506 (AGN33483.1) makes it 25 aa shorter than the products of *repJ* and PSA3335_1080; therefore, we used the product deduced from positions 872-1,729 of sequence accession no. CP003042, which contains a more likely start codon and that is the same size than RepJ from pPsv48C.
