# Supplementary material for: Plasmid Replicons from Pseudomonas Are Natural Chimeras of Functional, Exchangeable Modules
Source: Front Microbiol. 2017 Feb 13;8:190. doi: 10.3389/fmicb.2017.00190 (PMC5304414; doi:10.3389/fmicb.2017.00190)
Supplement: Supplementary file 2 [file Table2.PDF]

Table S2. Blastn comparison of the 600 nt situated 5' of the *repA* gene from *Pseudomonas fluorescens* R124 plasmid pMP-R124 (coordinates 43,195-43,794, accession no. JQ737005.1).[a]

Description	Replicon type	Total score (bits)	Query coverage	nt Identity	E value	Accession number
P. fluorescens L228, plasmid	RepA-PFP	616	85%	87%	2e-172	CP015640.1
P. fluorescens A506 plasmid pA506,	RepJ	499	85%	82%	4e-137	CP003042.1
P. syringae strain PT14 plasmid pPT14-32	RepA-PFP	479	80%	83%	4e-131	JQ418536.1
Uncultured bacterium plasmid Drgb7	RepA-RA2	221	79%	71%	2e-53	KT351738.1
P. aeruginosa PA7790 plasmid pPA7790	RepA-RA2	165	77%	69%	1e-36	CP015000.1
P. aeruginosa PSE305	RepA-RA2	165	77%	69%	1e-36	HG974234.1
P. balearica plasmid pKF707	RepA-RA2	131	78%	67%	2e-26	AP014863.1
P. alcaligenes plasmid pRA2,	RepA-RA2	127	66%	68%	3e-25	U88088.2

[a] The comparison was done using the blastn tool from the NCBI against the non-redundant nucleotide collection in December, 2016, with default parameters; shown are all hits with more than 65 % identity over more than 65 % of the query sequence. Rows with the same colour correspond to homologous replicons.
