# Supplementary material for: Plasmid Replicons from Pseudomonas Are Natural Chimeras of Functional, Exchangeable Modules
Source: Front Microbiol. 2017 Feb 13;8:190. doi: 10.3389/fmicb.2017.00190 (PMC5304414; doi:10.3389/fmicb.2017.00190)
Supplement: Supplementary file 3 [file Table3.pdf]

Table S3. Bacterial strains and plasmids used in this study.

Strain or plasmid	Host of isolation; description	Source or reference
**Bacteria**		
Escherichia coli		
DH10B	Δ(*mrr-hsdRMS-mcrB*) *deoR recA1 endA1 araD139* Δ(*ara*, *leu*)7697 *galU galK* λ^- *rpsL nupG*	(Grant et al., 1990)
GM2929	F-, *araC14*, *leuB6*, *fhuA13*, *lacY1*, *tsx-78*, *glnX44*, *galK2*, *galT22*, λ^-, *mcrA0*, *dcm-6*, *hisG4*, *rfbC1*, *rpsL136* (str^R), *dam-13::Tn9*, *xylA5*, *mtl-1*, *recF143*, *thiE1*, *mcrB9999*, *hsdR2*	(Palmer and Marinus, 1994)
Pseudomonas syringae		
pv. phaseolicola		
1448A	*Phaseolus vulgaris*; pathogen of bean, it contains two native plasmids (p1448A-A and p1448A-B).	(Joardar et al., 2005)
pv. savastanoi		
NCPPB 3335	*Olea europaea*; plant pathogen, causes aerial tumors on olive trees, it contains three native plasmids (pPsv48A, pPsv48B and pPsv48C)	National Collection of Plant Pathogenic Bacteria
UPN912	Derivative of strain NCPPB 3335 and cured of its three native plasmids	Maite Añorga, unpublished
pv. syringae		
B728a	*Phaseolus vulgaris*; pathogen of bean, plasmidless, Cu^r, Rif^r, Sm^r.	(Feil et al., 2005)
**Native plasmids**		
p1448A-A	RepA-PFP replicon; virulence plasmid from strain 1448A, 132 kb; CP000059.1	(Joardar et al., 2005)
p1448A-B	RepA-PFP replicon; from strain 1448A, 52 kb; CP000060.1	(Joardar et al., 2005)
pPsv48A	RepA-PFP replicon; virulence plasmid from strain NCPPB 3335, 78 kb; FR820585	(Bardaji et al., 2011)
pPsv48B	RepA-PFP replicon; from strain NCPPB 3335, 45 kb; FR820586	(Bardaji et al., 2011)
pPsv48C	Contains two replicons (RepA-PFP and RepJ); virulence plasmid from strain NCPPB 3335, 42 kb; FR820587	(Bardaji et al., 2011)
**Plasmids**		
pBBR1MCS-2	Broad host range cloning vector, 5.4 kb, Km^R	(Kovach et al., 1995)
pBlueScript II SK	Cloning vector, 2.96 kb, Amp^R	Stratagene
pK184	*E. coli* vector, does not replicate in *Pseudomonas*; 2.4 kb, Km^R	(Jobling and Holmes, 1990)

pKMAG	Vector derived from pK184, devoid of the P*lac* promoter and containing the transcriptional terminator and polylinker from pME6041; 2.6 kb, Km^R; KX714576	This work
pKMAG-C	pKMAG containing the minimal RepA-PFP replicon from pPsv48C, replicates in *E. coli* and in *Pseudomonas*; 4.3 kb, Km^R; KX714577	This work
pME6031	Cloning vector; 8.3 kb, Km^R	(Heeb et al., 2000)
pME6041	Cloning vector; 5.6 kb, Km^R	(Heeb et al., 2000)

References

Bardaji, L., Pérez-Martínez, I., Rodríguez-Moreno, L., Rodríguez-Palenzuela, P., Sundin, G.W., Ramos, C., et al. (2011). Sequence and role in virulence of the three plasmid complement of the model tumor-inducing bacterium *Pseudomonas savastanoi* pv. savastanoi NCPPB 3335. *PLoS ONE* 6, e25705. doi: 10.1371/journal.pone.0025705

Feil, H., Feil, W.S., Chain, P., Larimer, F., DiBartolo, G., Copeland, A., et al. (2005). Comparison of the complete genome sequences of *Pseudomonas syringae* pv. syringae B728a and pv. tomato DC3000. *Proc. Natl. Acad. Sci.* 102, 11064-11069. doi: 10.1073/pnas.0504930102

Grant, S.G., Jessee, J., Bloom, F.R., and Hanahan, D. (1990). Differential plasmid rescue from transgenic mouse DNAs into *Escherichia coli* methylation-restriction mutants. *Proc. Natl. Acad. Sci.* 87, 4645-4649

Heeb, S., Itoh, Y., Nishijyo, T., Schnider, U., Keel, C., Wade, J., et al. (2000). Small, stable shuttle vectors based on the minimal pVS1 replicon for use in gram-negative, plant-associated bacteria. *Mol. Plant-Microbe Interact.* 13, 232-237. doi: 10.1094/Mpmi.2000.13.2.232

Joardar, V., Lindeberg, M., Jackson, R.W., Selengut, J., Dodson, R., Brinkac, L.M., et al. (2005). Whole-genome sequence analysis of *Pseudomonas syringae* pv. phaseolicola 1448A reveals divergence among pathovars in genes involved in virulence and transposition. *J. Bacteriol.* 187, 6488-6498. doi: 10.1128/JB.187.18.6488-6498.2005

Jobling, M.G., and Holmes, R.K. (1990). Construction of vectors with the p15a replicon, kanamycin resistance, inducible *lacZ*α and pUC18 or pUC19 multiple cloning sites. *Nucleic Acids Res.* 18, 5315-5316. doi: 10.1093/nar/18.17.5315

Kovach, M.E., Elzer, P.H., Hill, D.S., Robertson, G.T., Farris, M.A., Roop II, R.M., et al. (1995). Four new derivatives of the broad-host-range cloning vector pBBR1MCS, carrying different antibiotic-resistance cassettes. *Gene* 166, 175-176. doi: 10.1016/0378-1119(95)00584-1

Palmer, B.R., and Marinus, M.G. (1994). The *dam* and *dcm* strains of *Escherichia coli*-a review. *Gene* 143, 1-12. doi: 10.1016/0378-1119(94)90597-5
