# Supplementary material for: Plasmid Replicons from Pseudomonas Are Natural Chimeras of Functional, Exchangeable Modules
Source: Front Microbiol. 2017 Feb 13;8:190. doi: 10.3389/fmicb.2017.00190 (PMC5304414; doi:10.3389/fmicb.2017.00190)
Supplement: Supplementary file 4 [file Image1.PDF]

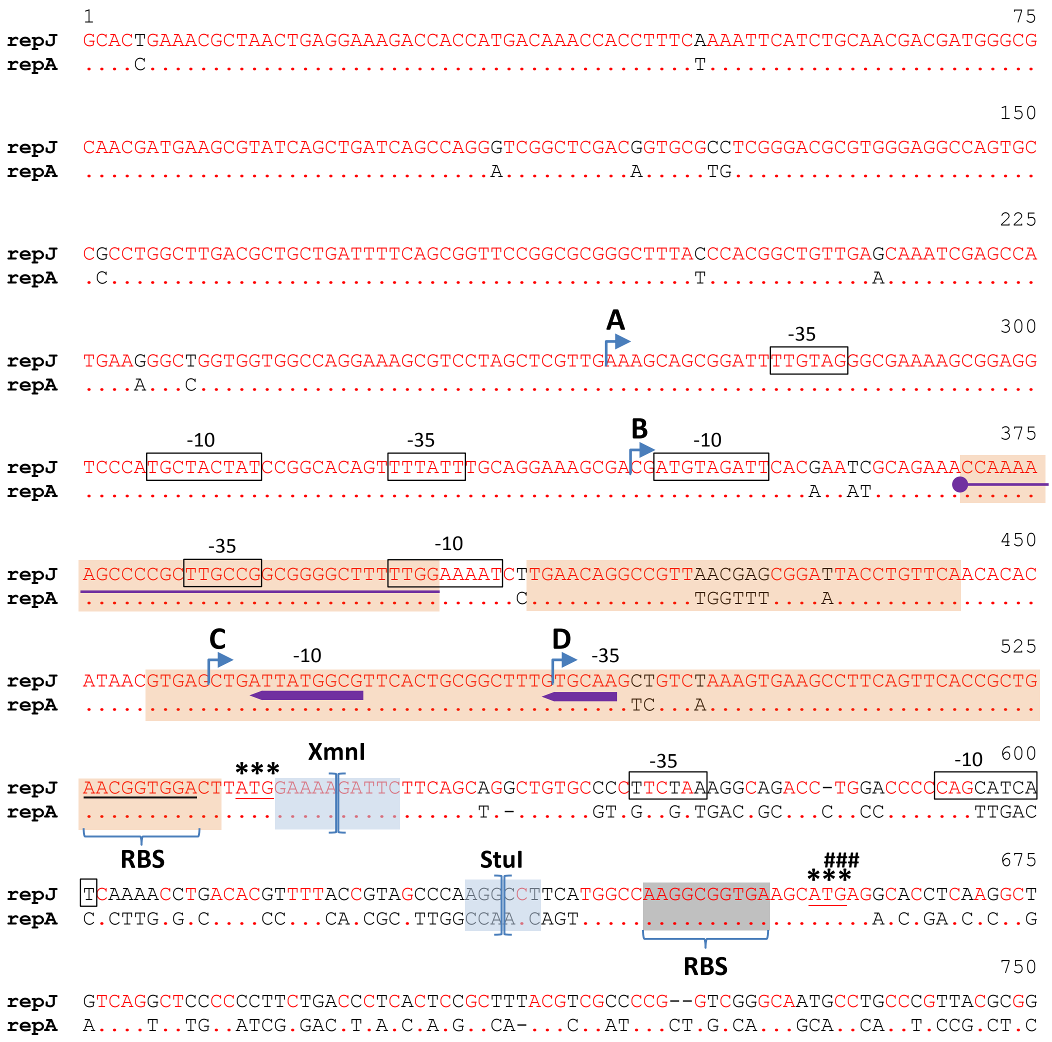

Figure S1. Global alignment of the REx-C modules preceding genes *repJ* and *repA* from plasmid pPsv48C. Sequences from coordinates 29,043-29,789 (*repJ*) and [41,488-42,103]-[1-92] (*repA*) from pPsv48C (accession no. FR820587) were aligned with Multalin, with identical positions in both sequences shown as red dots and mismatches as black nucleotides; dashes indicate gaps to maximize the alignments. Homology is lost before the first nucleotide shown here. Orange boxes correspond to stem-and-loop structures 1, 2 and 3, in this order; structure 3 is complex and can potentially fold in different ways. Angled arrows topped with letters indicate the position and start of primers used to define the minimal replicon, as depicted in Fig. 1. Blue boxes indicate restriction sites used for creating an in-frame deletion in *repI* and for constructing chimeric replicons. Asterisks and hashes indicate the putative start and stop codons, respectively, of both the leader peptide (*repI*) and the replication initiator (*repJ*) coding sequences; putative ribosome binding sites (RBS) are underlined for *repI* and boxed in grey for *repJ*. Boxes labelled as -35 and -10 are putative promoters; leftwards purple arrows indicate predicted -35 and -10 boxes of a putative antisense RNA promoter, with the predicted transcriptional terminator also indicated in purple. All promoters were predicted using BPROM.
