# Supplementary material for: Plasmid Replicons from Pseudomonas Are Natural Chimeras of Functional, Exchangeable Modules
Source: Front Microbiol. 2017 Feb 13;8:190. doi: 10.3389/fmicb.2017.00190 (PMC5304414; doi:10.3389/fmicb.2017.00190)
Supplement: Supplementary file 5 [file Image2.PDF]

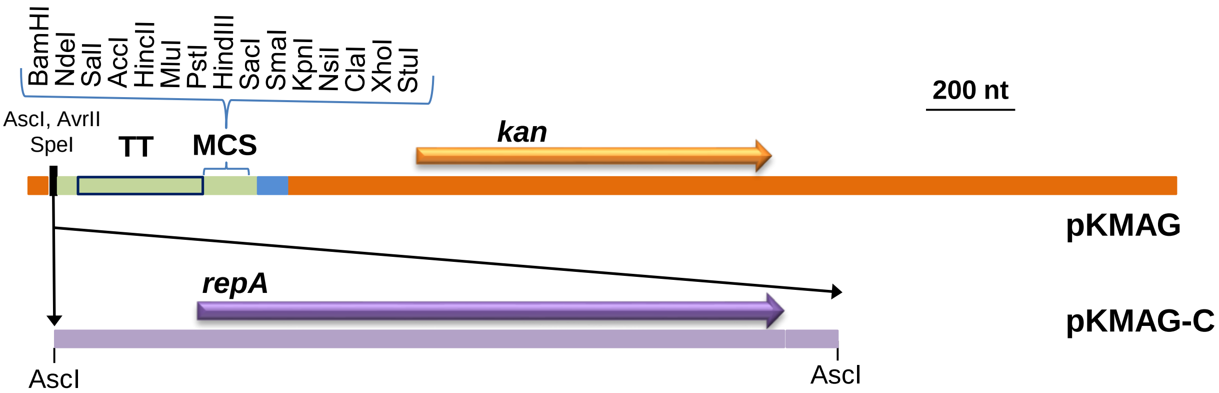

Figure S2. Schematic representation of pKMAG and pKMAG-C cloning vectors (accession numbers KX714576 and KX714577, respectively). Vectors were constructed by combining PCR amplification and ligation of DNA from diverse plasmids, as indicated by colors: green, from vector pME6041 (positions 3,809-4,255, accession no. AF118812); blue, from pGEM-T Easy; orange, from the *E. coli* vector pK184 (positions 71-2,064, accession no. U00800). Vector pKMAG-C contains the *repA* replicon from the native plasmid pPsv48C (positions 41,791-1,428, accession no. FR820587), cloned at the AscI site of pKMAG and allowing replication in *P. syringae*. The orange and purple arrows indicate the kanamycin resistance gene from pK184 and the *repA* gene from pPsv48C, respectively. All restriction sites shown in pKMAG are unique in the sequence. Sequences are drawn to scale; TT, transcriptional terminator; MCS, multiple cloning site.
