# Supplementary material for: Plasmid Replicons from Pseudomonas Are Natural Chimeras of Functional, Exchangeable Modules
Source: Front Microbiol. 2017 Feb 13;8:190. doi: 10.3389/fmicb.2017.00190 (PMC5304414; doi:10.3389/fmicb.2017.00190)
Supplement: Supplementary file 6 [file Image3.pdf]

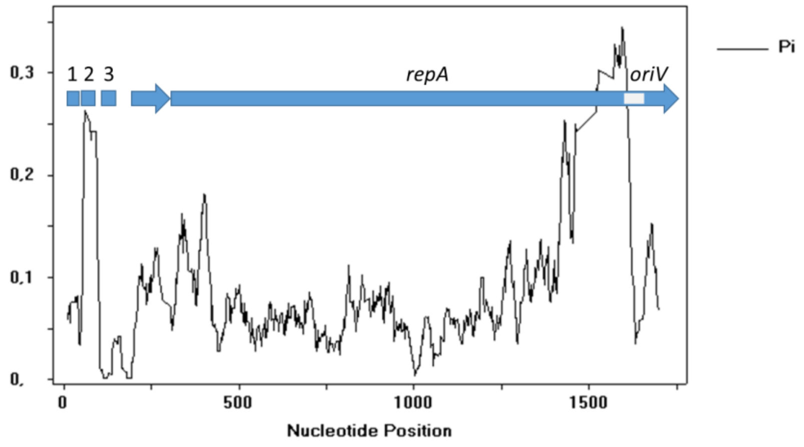

RepA-PFP replicon

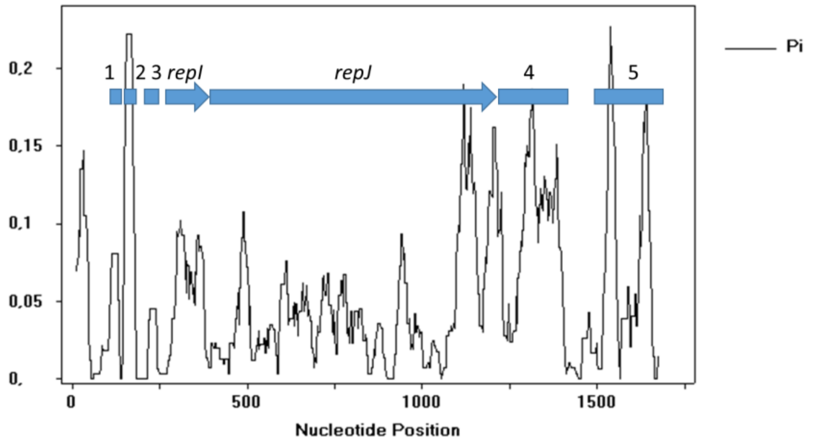

RepJ replicon

Figure S3. Sliding window analysis of nucleotide diversity (π) of RepA-PFP and RepJ replicons. Alignments consisted of 171 sequences (1,599 nt) for RepA-PFP and 23 sequences (1,688 nt) from clade I (see Fig. 2) for RepJ, all from strains of the *P. syringae* group. Nucleotide diversity was calculated using the program DnaSP v5.10.01 with a window length of 25 nt and a step of 2 nt and excluding all sites with gaps from the analysis. The approximate location of stem-and-loop structures (rectangles) and CDSs for the putative leader peptide (small arrows) and replication initiator proteins (large arrows) are shown; the origin of replication of RepA-PFP replicons is denoted as a white rectangle within the repA CDS.
