# Supplementary material for: Plasmid Replicons from Pseudomonas Are Natural Chimeras of Functional, Exchangeable Modules
Source: Front Microbiol. 2017 Feb 13;8:190. doi: 10.3389/fmicb.2017.00190 (PMC5304414; doi:10.3389/fmicb.2017.00190)
Supplement: Supplementary file 7 [file Image4.pdf]

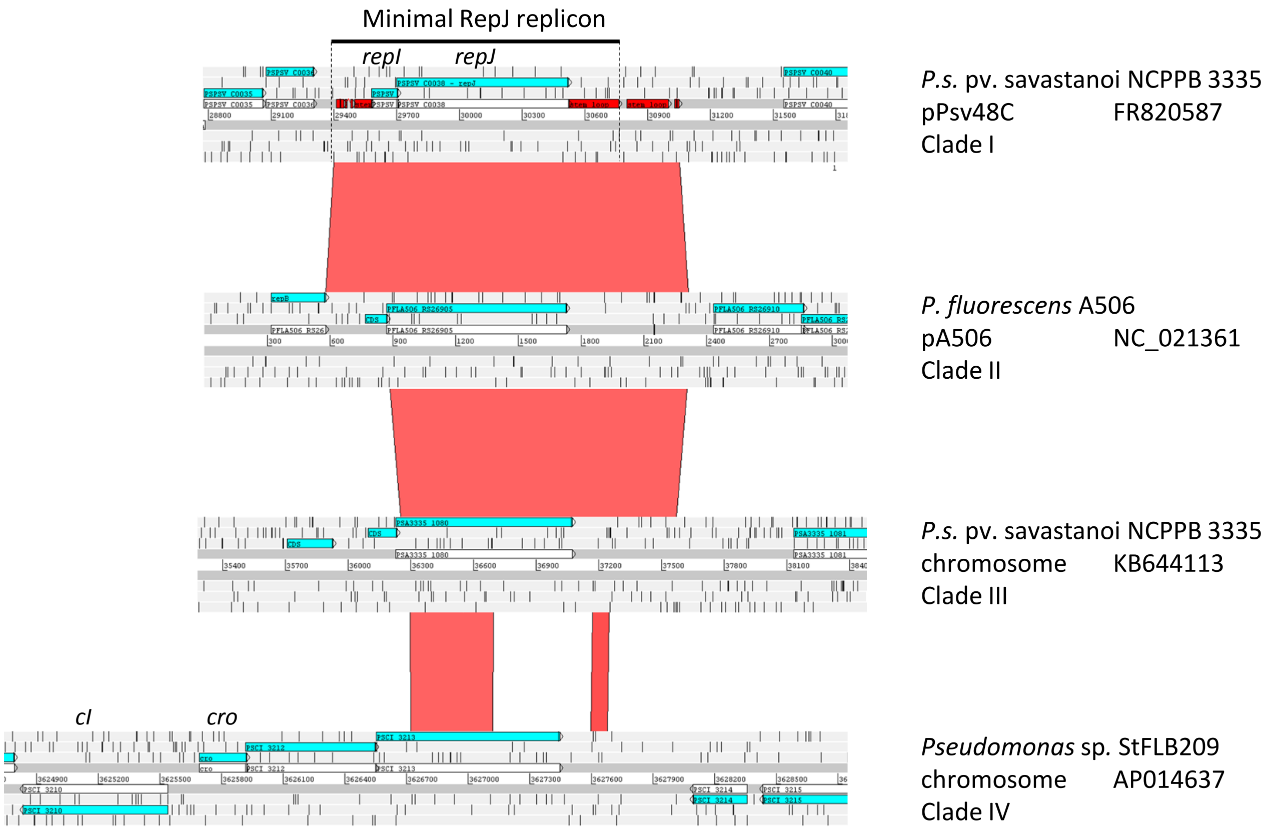

Figure S4. Genomic context of different RepJ homologs. Sequences are indicated by strain designation, genomic location and accession number. The extent of the minimal RepJ replicon (defined in Fig. 1) is indicated above the first sequence; stem-and-loop structures in the RepJ replicon from pPsv48C are shown as red boxes. Sequences were compared in pairs using blastn at the NCBI with default settings, except for a word size of 7 and a gap cost of 2/2 (existence/extension) to maximize alignment. Relevant areas were visualized using ACT; red shading connects homologous regions. In the lower sequence, *cI* and *cro* denote genes for putative repressor cI and regulatory Cro proteins, respectively.
