# Supplementary material for: Plasmid Replicons from Pseudomonas Are Natural Chimeras of Functional, Exchangeable Modules
Source: Front Microbiol. 2017 Feb 13;8:190. doi: 10.3389/fmicb.2017.00190 (PMC5304414; doi:10.3389/fmicb.2017.00190)
Supplement: Supplementary file 8 [file Image5.pdf]

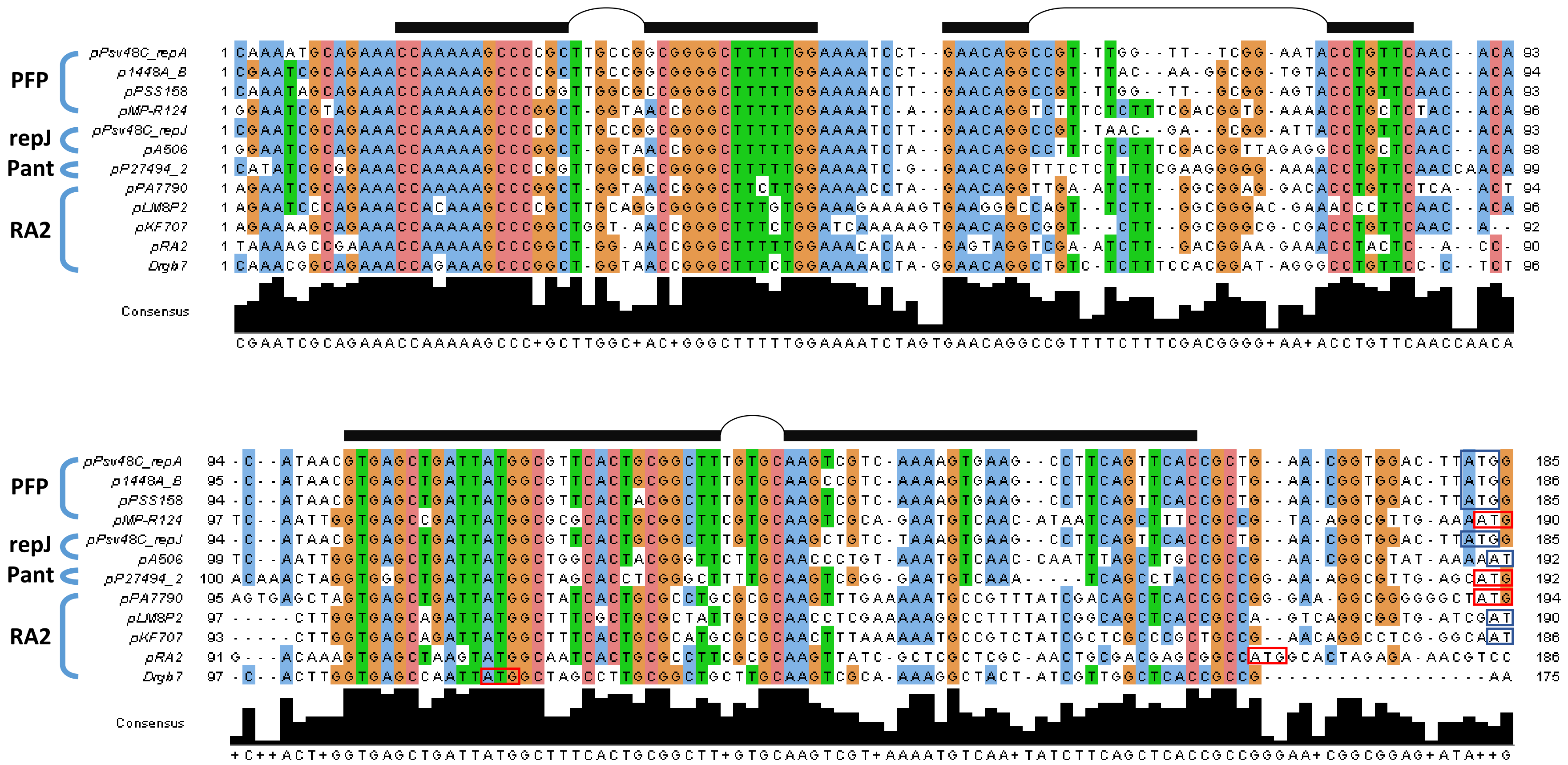

Figure S5. Sequence conservation of the REx-C modules from four replicon families. Alignment was done using the T-Coffee server at the EMBL-EBI website (http://www.ebi.ac.uk/Tools/msa/tcoffee/) with default parameters. Shown are partial sequences immediately preceding or overlapping the start of the *rep* genes from plasmids (accession no.:coordinates in parenthesis) pPsv48C (FR820587.1:41,804-41,988, *repA*, and 29,399-29,583, *repJ*); p1448A-B (CP000060.1:3-188); pPSS158 (CP005971.1:26,897-27,081); pMP-R124 (JQ737005.1:43,608-3); pA506 (CP003042.1:578-769); p27494_2 (CP015602.1:6,766-6,957); pPA7790 (CP015000.1:c46,674-46,481); pLM8P2 (KJ940994.1:22,563-2); pKF707 (AP014863.1:c44,123-43,928); pRA2 (U88088.2:c8,241-8,056), and Drgb7 (KT351738.1:c17,735-17,561). Sequences are grouped by groups of homology of their respective replication initiator proteins, indicated at left; nucleotides conserved in at least half of the sequences are highlighted in color. Blue boxes indicate the start codon of the putative leader peptide, and red boxes the start codon of the annotated *rep* gene. Bars above the sequence indicate stem-and-loop structures 1, 2 and 3, as discussed in the main text and Fig. S2.
