# Supplementary material for: Plasmid Replicons from Pseudomonas Are Natural Chimeras of Functional, Exchangeable Modules
Source: Front Microbiol. 2017 Feb 13;8:190. doi: 10.3389/fmicb.2017.00190 (PMC5304414; doi:10.3389/fmicb.2017.00190)
Supplement: Supplementary file 9 [file Image6.PDF]

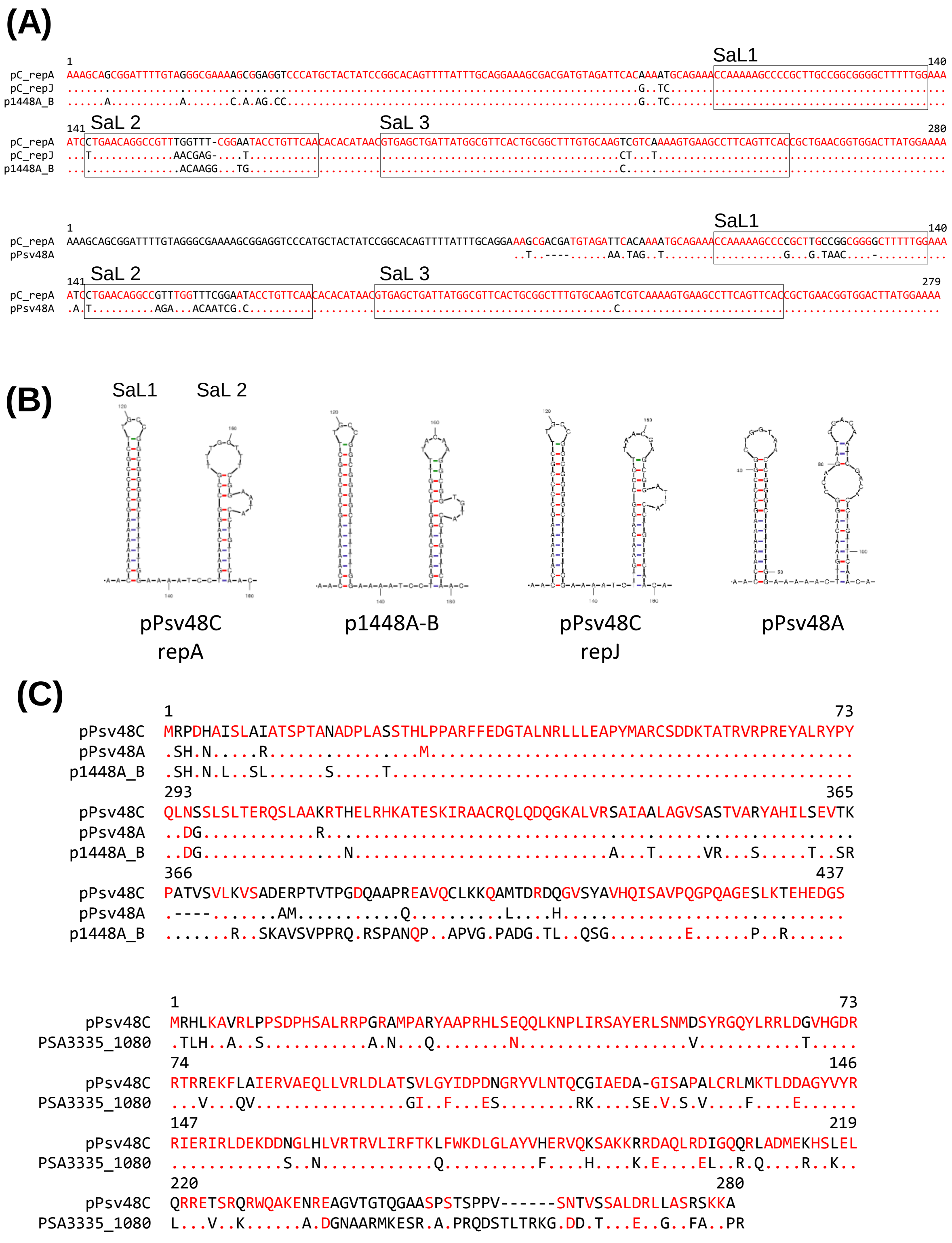

Figure S6. Sequence differences among the stem-and-loop structures and deduced products of *rep* genes used for the construction of chimeras. (A) Alignment with Multalin of the SaL fragments from the REx-C modules used for the construction of chimeras (see Fig. 4A), which end in the common XmnI site. Stem-and-loop structures 1, 2 and 3 are boxed. (B) Mfold predicted folding at 25 ºC of the sequences shown in A. (C) Alignment of the deduced products of replication initiators of the RepA-PFP (top) and the RepJ (bottom) replicons. For clarity, long stretches of sequence identity were removed from the RepA-PFP comparison. Residues identical to the topmost sequences are shown as dots and mismatches as black characters; dashes indicate gaps to maximize the alignments.
