# Supplementary material for: Plasmid Replicons from Pseudomonas Are Natural Chimeras of Functional, Exchangeable Modules
Source: Front Microbiol. 2017 Feb 13;8:190. doi: 10.3389/fmicb.2017.00190 (PMC5304414; doi:10.3389/fmicb.2017.00190)
Supplement: Supplementary file 10 [file Image7.PDF]

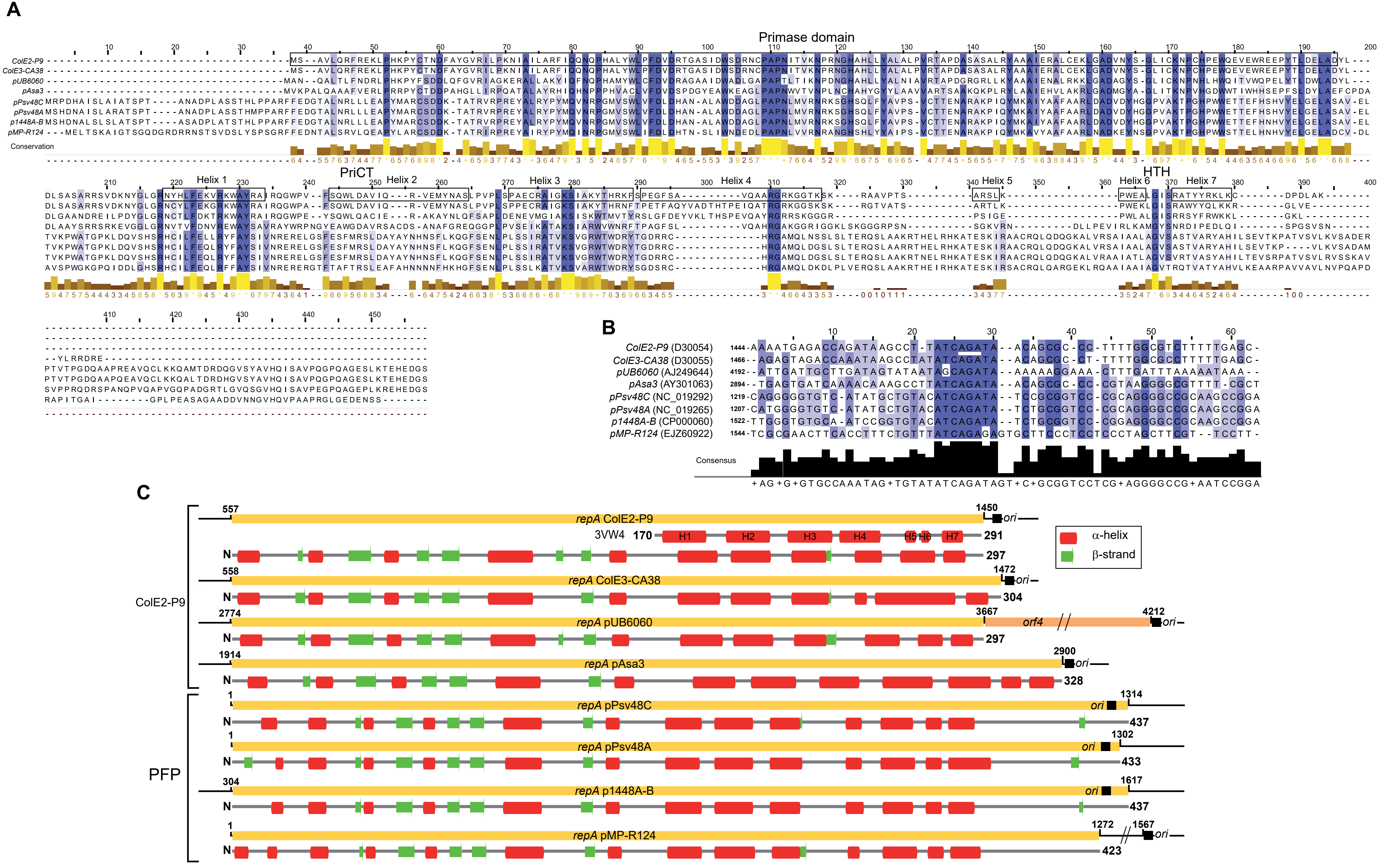
A
Primase domain
PriCT
Helix 1
Helix 2
Helix 3
Helix 4
Helix 5
Helix 6
Helix 7
HTH
ColE2-P9
ColE3-CA38
pUB6060
pAsa3
pPsv48C
pPsv48A
p1448A-B
pMP-R124
Conservation
B
ColE2-P9 (D30054)
ColE3-CA38 (D30055)
pUB6060 (AJ249644)
pAsa3 (AY301063)
pPsv48C (NC_019292)
pPsv48A (NC_019265)
p1448A-B (CP000060)
pMP-R124 (EJZ60922)
Consensus
C
ColE2-P9
PFP
repA ColE2-P9
3VW4
repA ColE3-CA38
repA pUB6060
orf4
repA pAsa3
repA pPsv48C
repA pPsv48A
repA p1448A-B
repA pMP-R124
ori
α-helix
β-strand

Figure S7

FIGURE S7. Comparison of Rep and *ori* sequences of various plasmids of the ColE2-P9 and PFP groups. A) Multiple sequence alignment performed with the Clustal Omega server in the EMBL-EBI website (https://www.ebi.ac.uk/Tools/msa/clustalo/) with default parameters. Shown are sequences of the replication proteins from plasmids (accession no. in parenthesis) ColE2-P9 (Q51629), ColE3-CA38 (Q51633), pUB6060 (Q9RLE8), pAsa3 (Q7X2E2), pPsc48C (YP_006962014.1), pPsv48A (YP_006961523.1), p1448-B (AAZ38092.1), pMP-R124 (AFS51677.1). The predicted primase domain as well the position of the helixes 1 to 7 comprising the PriCT and HTH domains is indicated on the ColE2-P9 Rep sequence. Amino acid residues conserved in at least half of the sequences are highlighted in colour. The histogram below the sequences indicates the degree of conservation of the physico-chemical properties in the alignment. Each column is scored with a numerical index although conserved columns are indicated by '*' and columns with mutations where all properties are conserved are marked with a '+'. Of note, key residues in the turn of the potential HTH DNA binding motif are conserved. B) Comparison of the putative origin sequences of various plasmids from the PFP group with the origin sequences of ColE2 related plasmids (the four plasmids at the top). The position number in the database sequence (accession number shown in parenthesis) of the nucleotide at the left end of each sequence included in the analysis is indicated. Nucleotides conserved in at least half of the sequences are highlighted in colour. A line below the sequence of ColE2 *ori* indicates the position of the primer RNA (AGA). C) Predicted secondary structure of the Rep proteins analysed in panel A. The top horizontal line shows the coordinates in the plasmid of the gene encoding the Rep protein as well as the position of the confirmed or putative origin of replication. The bottom horizontal line shows the result of the secondary structure prediction performed with the Jpred server in the http://www.compbio.dundee.ac.uk/jpred/ website. The amino-terminal end (N) and the number of amino acids are indicated for each protein analysed.
